# Gene Expression Profile of Human Cytokines in Response to *Burkholderia pseudomallei* Infection

**DOI:** 10.1128/mSphere.00121-17

**Published:** 2017-04-19

**Authors:** Shivankari Krishnananthasivam, Harindra Darshana Sathkumara, Enoka Corea, Mohan Natesan, Aruna Dharshan De Silva

**Affiliations:** a Genetech Research Institute, Colombo, Sri Lanka; b Department of Microbiology, Faculty of Medicine, University of Colombo, Colombo, Sri Lanka; c Molecular and Translational Sciences, United States Army Medical Research Institute of Infectious Diseases, Frederick, Maryland, USA; d Division of Vaccine Discovery, La Jolla Institute of Allergy and Immunology, La Jolla, California, USA; University of Rochester

**Keywords:** *Burkholderia pseudomallei*, gene expression profiling, host immune responses, human host cytokine cascade, melioidosis

## Abstract

Melioidosis is an underreported infectious disease, caused by the Gram-negative bacterium *Burkholderia pseudomallei*. Understanding the disease susceptibility and pathogenesis is crucial for developing newer diagnostic and therapeutic strategies for this life-threatening infection. In this study, we aimed to analyze the gene expression levels of important cytokines in melioidosis patients and establish useful correlates with disease biomarkers compared to cases of sepsis infection caused by other pathogens and healthy individuals. A Qiagen common human cytokines array profiling the gene expression of 84 important cytokines by real-time quantitative PCR (RT-qPCR) was used. We analyzed 26 melioidosis cases, 5 healthy controls, and 10 cases of sepsis infection caused by other pathogens. Our results showed consistently upregulated expression of interleukins (IL) interleukin-4 (IL-4), interleukin-17 alpha (IL-17A), IL-23A, and IL-24, interferons (IFN) interferon alpha 1 (IFNA1) and interferon beta 1 (IFNB1), tumor necrosis factor (TNF) superfamily 4 (TNFSF4), transforming growth factor (TGF) superfamily, bone morphogenetic proteins 3 and 6 (BMP3 and BMP6), transforming growth factor beta 1 (TGFB1), and other growth factors, including macrophage colony-stimulating factor (M-CSF), C-fos-induced growth factor (FIGF), and platelet-derived growth factor alpha (PDGFA) polypeptide, in melioidosis patients compared to their expression in other sepsis cases, irrespective of comorbidities, duration of fever/clinical symptoms, and antibiotic treatment. Our findings indicate a dominant Th2- and Th17-type-cytokine response, suggesting that their dysregulation at initial stages of infection may play an important role in disease pathogenesis. IL-1A, interleukin-1 beta (IL-1B), and IL-8 were significantly downregulated in septicemic melioidosis patients compared to their expression in other sepsis cases. These differentially expressed genes may serve as biomarkers for melioidosis diagnosis and targets for therapeutic intervention and may help us understand immune response mechanisms.

**IMPORTANCE** Melioidosis is a life-threatening infectious disease caused by a soil-associated Gram-negative bacterium, *B. pseudomallei*. Melioidosis is endemic in Southeast Asia and northern Australia; however, the global distribution of *B. pseudomallei* and the disease burden of melioidosisis are still poorly understood. Melioidosis is difficult to treat, as *B. pseudomallei* is intrinsically resistant to many antibiotics and requires a long course of antibiotic treatment. The mortality rates remain high in areas of endemicity, with reoccurrence being common. Therefore, it is imperative to diagnose the disease at an early stage and provide vital clinical care to reduce the mortality rate. With limitations in treatment and lack of a vaccine, it is crucial to study the immune response mechanisms to this infection to get a better understanding of disease susceptibility and pathogenesis. Therefore, this study aimed to analyze the gene expression levels of important cytokines to establish useful correlations for diagnostic and therapeutic purposes.

## Abstract (precis)

Melioidosis is a life-threatening infectious disease caused by a soil-associated Gram-negative bacterium, *B. pseudomallei*. Melioidosis is endemic in Southeast Asia and northern Australia; however, the global distribution of *B. pseudomallei* and the disease burden of melioidosisis are still poorly understood. Melioidosis is difficult to treat, as *B. pseudomallei* is intrinsically resistant to many antibiotics and requires a long course of antibiotic treatment. The mortality rates remain high in areas of endemicity, with reoccurrence being common. Therefore, it is imperative to diagnose the disease at an early stage and provide vital clinical care to reduce the mortality rate. With limitations in treatment and lack of a vaccine, it is crucial to study the immune response mechanisms to this infection to get a better understanding of disease susceptibility and pathogenesis. Therefore, this study aimed to analyze the gene expression levels of important cytokines to establish useful correlations for diagnostic and therapeutic purposes.

## INTRODUCTION

Melioidosis is a life-threatening infectious disease that is endemic in Southeast Asia and northern Australia (1). A recent report estimates the melioidosis disease burden to be 165,000 cases per year (2). Lack of awareness of melioidosis disease among physicians and lack of diagnostic methods contribute to underreporting in many countries where it is endemic. Infection is suspected to be acquired mainly via the skin during exposure to soil and contaminated water. Nevertheless, inhalation of aerosolized bacteria during extreme weather events like rainfall and storms has also been reported (2, 3). The disease is strongly associated with comorbidities such as diabetes mellitus, chronic kidney disease, thalassemia, immunosuppression, and excessive alcohol intake (1, 4, 5). A broad spectrum of clinical presentations, ranging from acute fulminant septicemia to chronic localized abscesses, are reported for melioidosis (5). Early diagnosis and appropriate antibiotic treatment play a crucial role in preventing mortality and recurrence. The advancement of new immunodiagnostic methods and therapeutic strategies is important for disease management of melioidosis, given the lack of vaccines and limitations in drug treatment (3).

Studying the host immune responses to infection is crucial for understanding disease susceptibility and pathogenesis and immune correlates of protection (3). Cytokines are vital immune modulators that regulate and determine the nature of immune responses to an infection (6). The activation of leukocytes and cytokine networks is a prominent feature of inflammation and the septic response (7). Pro- and anti-inflammatory cytokines play a critical role in regulating overall immune responses and in establishing homeostasis, and their dysregulation is instrumental in triggering disease progression and severity (8). Hence, a detailed study of the cytokine cascade events at the transcriptome level during an infection is useful to understand disease pathogenesis and susceptibility. Although cytokine cascade events following *Burkholderia pseudomallei* infection have been studied in several animal models (7, 9–13), data on human host mRNA expression levels of cytokines are limited. Proinflammatory cytokines like interleukin-8 (IL-8), IL-6, IL-12, IL-18, IL-15, interferon gamma (IFN-γ), tumor necrosis factor alpha (TNF-α), and IL-1β, anti-inflammatory cytokines like IL-4, and several other chemokines have been implicated in disease outcome during the early acute phase of *B. pseudomallei* infection (7, 14–16). While individual cytokines have been investigated in previous studies, the profiling of entire cytokine networks is necessary to comprehensively understand specific immune response pathways and, thereby, the pathophysiology of melioidosis. Such a profile may also help identify disease biomarkers with therapeutic implications.

We have successfully established a nationwide surveillance system in Sri Lanka that has resulted in finding more confirmed cases of melioidosis (unpublished data). In this study, we aimed to analyze the gene expression profiles of 84 important human cytokines (see Table S1 in the supplemental material) in Sri Lankan melioidosis patients to further understand the immune response mechanisms during melioidosis and establish useful correlates with disease biomarkers.

10.1128/mSphere.00121-17.1 TABLE S1  List of 84 gene targets investigated in the cytokine expression profiling study. Download TABLE S1, PDF file, 0.1 MB. Copyright © 2017 Krishnananthasivam et al. 2017 Krishnananthasivam et al. This content is distributed under the terms of the Creative Commons Attribution 4.0 International license.

## RESULTS AND DISCUSSION

A total of 26 cases of melioidosis were analyzed, of which 23 were confirmed cases (culture positive) and 3 were probable cases (high antibody titer positive). A majority (*n* = 23) of melioidosis cases had associated comorbidities, and diabetes was the most common comorbidity (*n* = 17) in this study. Out of 23 confirmed cases of melioidosis, 16 were classified as septicemic or bacteremic.

**FIG 1:**
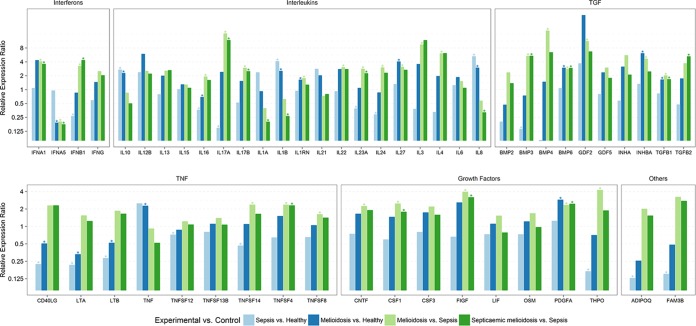
Relative differential gene expression of cytokines in melioidosis patients compared to the expression in patients with sepsis infection due to other pathogens and healthy negative controls. Significant relative gene expression changes in PBMCs from melioidosis patients (*n* = 26) and septicemic melioidosis patients (*n* = 16) compared to the expression in sepsis controls (*n* = 10) and healthy controls (*n* = 5) are shown. Expression levels were normalized against beta actin as the reference housekeeping gene. A relative expression ratio of >1.5 is considered to show upregulation, and a relative expression ratio of ≤0.5 is considered to show downregulation. *, relative expression ratio is significantly different (*P* < 0.05).

The differential pattern of the expression of interleukins, interferons, TNF superfamily, TGF superfamily, and other growth factors in melioidosis patients compared to their expression in other bacterial sepsis infection cases and healthy controls was significant. Adiponectin, C1Q, collagen domain containing (ADIPOQ), and family with sequence similarity 3, member B (FAM3B), were significantly downregulated in other bacterial sepsis infection cases compared to their expression in healthy controls (Fig. 1; see also Table S4 in the supplemental material).

### Gene expression profile of interleukins.

Our study reveals upregulated expression of IL-10, interleukin-1 beta (IL-1B), IL-1 receptor antagonist (IL-1RN), IL-27, and IL-8 in melioidosis patients compared to their expression in healthy controls (Table 1 and Fig. 1; see also Table S3 in the supplemental material). This is in agreement with a study by Wiersinga et al. reporting increased mRNA expression of inflammatory response genes like IL-1β, IL-6, IL-15, IL-10, IL-4, IFN-γ, and TNF-α in melioidosis patients compared to their expression in healthy controls (7). In our study, IL-16, interleukin-17 alpha (IL-17A), IL-23A, and IL-24 were downregulated while IL-10, IL-1B, and IL-8 were upregulated in other bacterial sepsis infection cases compared to their expression in healthy controls (Fig. 1; see also Table S4). IL-16, IL-17A, IL-17B, IL-1RN, IL-22, IL-23A, IL-24, IL-27, IL-3, and IL-4 were all upregulated in melioidosis patients compared to their expression in other bacterial sepsis infection cases (Table 2 and Fig. 1; see also Table S2). IL-17A, IL-3, and IL-4 showed particularly high levels of gene expression. Previously, the expression profiles of interleukins in response to *B. pseudomallei* infection have been extensively studied in several animal models, showing upregulated expression of interleukins like IL-1β, IL-6, IL-10, and IL-12 within 72 h of infection (9–13, 15). Elevated levels of expression of IL-6, IL-8, IL-12, IL-15, and IL-18 were also observed in the plasma of melioidosis patients (15, 16).

10.1128/mSphere.00121-17.2 TABLE S2  Relative expression ratios of 84 investigated gene targets in PBMCs of melioidosis patients (*n* = 26) compared to PBMCs of other sepsis cases (*n* = 10). Shading indicates statistically significant differential gene expression at a *P* value of <0.05. Download TABLE S2, PDF file, 0.1 MB. Copyright © 2017 Krishnananthasivam et al. 2017 Krishnananthasivam et al. This content is distributed under the terms of the Creative Commons Attribution 4.0 International license.

10.1128/mSphere.00121-17.3 TABLE S3  Relative expression ratios of 84 investigated gene targets in PBMCs of melioidosis patients (*n* = 26) compared to PBMCs of healthy controls (*n* = 5). Shading indicates statistically significant differential gene expression at a *P* value of <0.05. Download TABLE S3, PDF file, 0.1 MB. Copyright © 2017 Krishnananthasivam et al. 2017 Krishnananthasivam et al. This content is distributed under the terms of the Creative Commons Attribution 4.0 International license.

10.1128/mSphere.00121-17.4 TABLE S4  Relative expression ratios of 84 investigated gene targets in PBMCs of other sepsis infection cases (*n* = 10) compared to PBMCs of healthy controls (*n* = 5). Shading indicates statistically significant differential gene expression at a *P* value of <0.05. Download TABLE S4, PDF file, 0.1 MB. Copyright © 2017 Krishnananthasivam et al. 2017 Krishnananthasivam et al. This content is distributed under the terms of the Creative Commons Attribution 4.0 International license.

**TABLE 1:** Cytokines showing significant differential gene expression in PBMCs of melioidosis patients compared to their expression in healthy negative controls^a^

Gene target	Description	Relative expression ratio (95% CI)^b^	*P* value^c^
*IL-1B*	Interleukin-1 beta	2.504 (1.229–5.100)	0.0135
*IL-1RN*	Interleukin-1 receptor antagonist	1.62 (1.023–2.564)	0.0403
*IL-8*	Interleukin-8	2.953 (1.394–6.257)	0.0062
*IL-10*	Interleukin-10	2.257 (1.180–4.319)	0.0158
*IL-27*	Interleukin-27	4.022 (1.632–9.915)	0.0039
*INFA5*	Interferon alpha 5	0.189 (0.037–0.961)	0.0454
*TNF*	Tumor necrosis factor	2.248 (1.082–4.670)	0.0315
*CD40LG*	CD40 ligand	0.502 (0.336–0.752)	0.002
*LTA*	Lymphotoxin alpha	0.327 (0.190–0.565)	0.0003
*BMP6*	Bone morphogenetic protein 6	2.946 (1.470–5.904)	0.007
*INHBA*	Inhibin beta A	6.07 (2.652–13.891)	0.0002
*TGFB1*	Transforming growth factor beta 1	1.634 (1.121–2.383)	0.0126
*PDGFA*	Platelet-derived growth factor alpha polypeptide	2.86 (1.444–5.667)	0.0066

a PBMCs, peripheral blood mononuclear cells. *n* = 26 melioidosis patients; *n* = 5 healthy negative controls.

b A relative expression ratio of >1.5 indicates upregulation, and a relative expression ratio of ≤0.5 indicates downregulation in the experimental group compared to the expression in the control group. CI, confidence interval.

c Gene targets are considered to show significant differential expression at a *P* value of <0.05.

**TABLE 2:** Cytokines showing significant differential gene expression in PBMCs of melioidosis patients compared to their expression in other sepsis cases^a^

Gene target	Description	Relative expression ratio (95% CI)^b^	*P* value^c^
*IL-3*	Interleukin-3	9.38 (1.773–49.626)	0.0107
*IL-4*	Interleukin-4	6.024 (1.153–31.479)	0.0344
*IL-16*	Interleukin-16	1.896 (1.152–3.121)	0.0157
*IL-17A*	Interleukin-17 alpha	16.32 (3.193–83.421)	0.0017
*IL-17B*	Interleukin-17 beta	2.939 (1.486–5.811)	0.003
*IL-1RN*	Interleukin-1 receptor antagonist	1.747 (1.091–2.796)	0.0216
*IL-22*	Interleukin-22	3.022 (1.207–7.565)	0.0206
*IL-23A*	Interleukin-23 alpha	2.792 (1.329–5.866)	0.0092
*IL-24*	Interleukin-24	2.991 (1.240–7.214)	0.0173
*IL-27*	Interleukin-27	3.089 (1.203–7.932)	0.0206
*INFA1*	Interferon alpha 1	4.034 (1.358–11.984)	0.014
*INFA5*	Interferon alpha 5	0.2 (0.057–0.704)	0.0152
*INFB1*	Interferon beta 1	3.206 (1.056–9.735)	0.0407
*TNFSF4*	Tumor necrosis factor superfamily 4	2.349 (1.167–4.728)	0.0202
*TNFSF8*	Tumor necrosis factor superfamily 8	1.606 (1.004–2.571)	0.0484
*TNFSF14*	Tumor necrosis factor superfamily 14	2.353 (1.171–4.728)	0.0186
*BMP3*	Bone morphogenetic protein 3	5.305 (2.319–12.135)	0.0003
*BMP4*	Bone morphogenetic protein 4	18.765 (1.479–238.054)	0.0271
*BMP6*	Bone morphogenetic protein 6	2.776 (1.214–6.344)	0.0192
*GDF2*	Growth differentiation factor 2	11.112 (1.105–111.704)	0.0421
*INHBA*	Inhibin beta A	4.635 (1.205–17.822)	0.0282
*TGFB1*	Transforming growth factor beta 1	2.006 (1.374–2.931)	0.0007
*PDGFA*	Platelet-derived growth factor alpha polypeptide	2.317 (1.065–5.038)	0.0357
*THPO*	Thrombopoietin	4.213 (1.042–17.040)	0.0441
*CNTF*	Ciliary neurotrophic factor	2.222 (1.023–4.829)	0.0441
*CSF1*	Colony-stimulating factor 1	2.456 (1.451–4.156)	0.0017
*FIGF*	C-fos-induced growth factor	3.912 (1.561–9.802)	0.0049

a PBMCs, peripheral blood mononuclear cells. *n* = 26 melioidosis patients; *n* = 10 other sepsis cases.

b A relative expression ratio of >1.5 indicates upregulation, and a relative expression ratio of ≤0.5 indicates downregulation in the experimental group compared to the expression in the control group. CI, confidence interval.

c Gene targets are considered to show significant differential expression at a *P* value of <0.05.

**FIG 2:**
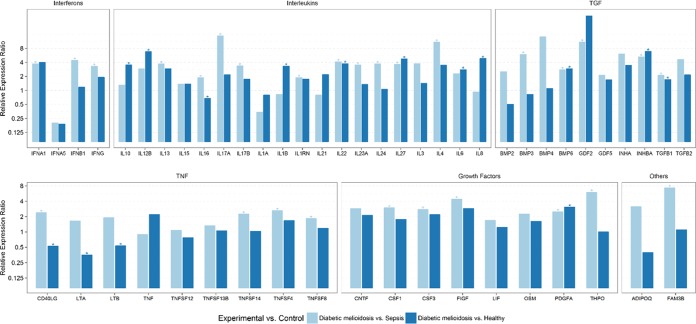
Relative differential gene expression of cytokines in diabetic melioidosis patients compared to the expression in patients with sepsis infection due to other pathogens and healthy negative controls. Relative gene expression in PBMCs from diabetic melioidosis patients (*n* = 17) and from sepsis controls (*n* = 10) and healthy controls (*n* = 5) is shown. Expression levels were normalized against beta actin as the reference housekeeping gene. A relative expression ratio of >1.5 is considered to show upregulation, and a relative expression ratio of ≤0.5 is considered to show downregulation. *, relative expression ratio is significantly different (*P* < 0.05).

In particular, IL-17A, a proinflammatory cytokine that mediates inflammatory responses and induces the production of other cytokines, is expressed at very high levels in melioidosis patients (including septicemic and diabetic cohorts) compared to its expression in other sepsis infections (Fig. 1 to 2; see also Tables S5 and S6 in the supplemental material). Additionally, IL-22, which is regarded as a Th17 cytokine, also shows upregulated expression in melioidosis patients compared to its expression in other sepsis cases. IL-17 and other Th17 cytokines are linked to the response against extracellular bacteria, as well as the pathogenesis of diverse autoimmune and inflammatory diseases, as their dysregulated expression can lead to uncontrolled inflammatory responses (17, 18). IL-17 is also implicated in excessive tissue damage by stimulating the production of many other cytokines, including granulocyte-colony-stimulating factor (G-CSF), granulocyte-macrophage colony stimulating factor (GM-CSF), transforming growth factor beta (TGF-β), and TNF-α, thus contributing to inflammatory pathology (8). IL-23, a key mediator of inflammation, has also been reported to show upregulated mRNA expression during *B. pseudomallei* infection, implicating its role in pathogenic host immune responses (19). Anti-IL-17 and anti-IL-23 antibodies have been shown to be effective in several immune-mediated inflammatory diseases (18, 20). IL-27, implicated in regulating B and T cell activity, has been reported to be significantly elevated in melioidosis patients compared to its levels in healthy controls, and overproduction of IL-27 plays a major role in the pathogenesis of sepsis and shock (21). IL-27 has also been identified as a potential sepsis biomarker and a candidate for successful therapeutic intervention (22, 23). As our results show consistently upregulated expression of IL-17, IL-23, and IL-27, their role in melioidosis disease progression and therapeutic use should be further investigated.

10.1128/mSphere.00121-17.5 TABLE S5  Relative expression ratios of 84 investigated gene targets in PBMCs of septicemic melioidosis cases (*n* = 16) compared to PBMCs of other sepsis cases (*n* = 10). Shading indicates statistically significant differential gene expression at a *P* value of <0.05. Download TABLE S5, PDF file, 0.1 MB. Copyright © 2017 Krishnananthasivam et al. 2017 Krishnananthasivam et al. This content is distributed under the terms of the Creative Commons Attribution 4.0 International license.

10.1128/mSphere.00121-17.6 TABLE S6  Relative expression ratios of 84 investigated gene targets in PBMCs of diabetic melioidosis cases (*n* = 17) compared to PBMCs of other sepsis cases (*n* = 10) and healthy controls (*n* = 5). Shading indicates statistically significant differential gene expression at a *P* value of <0.05. Download TABLE S6, PDF file, 0.1 MB. Copyright © 2017 Krishnananthasivam et al. 2017 Krishnananthasivam et al. This content is distributed under the terms of the Creative Commons Attribution 4.0 International license.

Our study revealed greater than 3-fold upregulation of IL-4, IL-13, IL-17A, IL-17B, IL-22, IL-23A, IL-24, and IL-27 in the diabetic melioidosis cohort (*n* = 17) compared to their expression in other bacterial sepsis cases (Fig. 2; see also Table S6 in the supplemental material). Diabetes, a risk factor for infectious diseases, may play a role in neutrophil and T cell dysfunction, possibly mediated by altered glucose metabolism and oxidative stress (24). Studies of melioidosis infection in diabetic cohorts (mice and human) show excessive neutrophil infiltration and impaired inflammatory and Th1 cytokine responses, leading to increased susceptibility of diabetic individuals to melioidosis (10, 25). IL-4, a key regulator of humoral and adaptive immunity, functions as an anti-inflammatory cytokine that decreases the production of Th1 cells and related proinflammatory cytokines. Our findings show upregulation of IL-4 and the closely related anti-inflammatory cytokine IL-13 in the melioidosis cohort compared to their expression in other sepsis cases, which is suggestive of inflammatory responses being dysregulated. Upregulated IL-4 expression has been reported in melioidosis patients and acute melioidosis animal models (7, 9).

**FIG 3:**
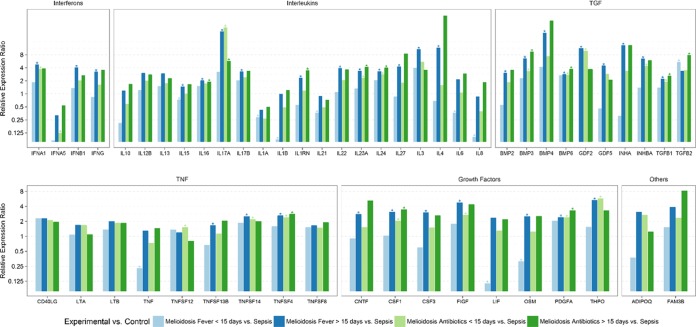
Relative differential gene expression of cytokines in melioidosis patients with respect to duration of fever/clinical symptoms and antibiotic treatment compared to their expression in patients with sepsis infection due to other pathogens. Relative gene expression in PBMCs from melioidosis patients with ≤15 days of fever (*n* = 5), melioidosis patients with >15 days of fever (*n* = 21), melioidosis patients with ≤15 days of treatment with antibiotics (*n* = 14), and melioidosis patients with >15 days of treatment with antibiotics (*n* = 8) compared to sepsis controls (*n* = 10) is shown. Expression levels were normalized against beta actin as the reference housekeeping gene. A relative expression ratio of >1.5 is considered to show upregulation, and a relative expression ratio of ≤0.5 is considered to show downregulation. *, relative expression ratio is significantly different (*P* < 0.05).

Our findings also show significant upregulation of IL-17A, IL-17B, and IL-23A, whereas IL-1A, IL-1B, and IL-8 were downregulated in the septicemic melioidosis cohort (*n* = 16) compared to their expression in other sepsis cases (Fig. 1; see also Table S5 in the supplemental material). Downregulation of IL-1A, IL-1B, IL-6, IL-8, and IL-21 in the early-acute-phase melioidosis cohort (<15 days fever/clinical symptoms) compared to their expression in other sepsis cases (Fig. 3; see also Table S7) was also seen, indicating that IL-1A, IL-1B, and IL-8 are potential markers during the early stages of inflammation and are correlated with disease severity. A study using a human lung epithelial cell line showed that IL-8 production was lower upon *B. pseudomallei* infection than in cells infected with other Gram-negative bacteria, which correlates with our findings (14). The association of increased IL-6 and IL-8 plasma concentrations with disease severity and mortality has also been reported (15). It has been reported that reduced immunity in melioidosis patients, correlating to mortality, is associated with upregulated IL-1R-associated kinase M expression leading to a strong decrease in the capacity to release proinflammatory cytokines like IL-1B, TNF-α, and IL-8 (26). Downregulation of IL-1B upon *B. pseudomallei* infection compared with its expression upon avirulent *Burkholderia thailandensis* infection in lung epithelial cells has also been reported, suggesting host response evasion (27). In our findings, we also see upregulation of interleukin receptor antagonist (ILRN), a natural inhibitor of the proinflammatory effects of IL-1A and IL-1B, in melioidosis patients compared to its expression in other bacterial sepsis cases. Thus, IL-1 and IL-8, which are key mediators of inflammation and early innate immune responses, may serve as candidate early diagnostic markers and indicators of disease severity.

10.1128/mSphere.00121-17.7 TABLE S7  Relative expression ratios of 84 investigated gene targets in PBMCs of melioidosis cases with ≤15 days of fever/clinical symptoms duration (*n* = 5) and >15 days fever/clinical symptoms duration (*n* = 21) compared to PBMCs of other sepsis cases (*n* = 10). Shading indicates statistically significant differential gene expression at a *P* value of <0.05. Download TABLE S7, PDF file, 0.2 MB. Copyright © 2017 Krishnananthasivam et al. 2017 Krishnananthasivam et al. This content is distributed under the terms of the Creative Commons Attribution 4.0 International license.

Our findings reveal an upregulated Th2 and Th17 cytokine response and a downregulated Th1 cytokine response, with associated comorbidities such as diabetes playing a key role in pathogenesis and severity through dysregulated cytokine responses.

### Gene expression profile of interferons.

Interferon alpha 5 (IFNA5) was downregulated in melioidosis patients compared to its expression in healthy controls (Table 1 and Fig. 1; see also Table S3 in the supplemental material). Interferon beta 1 (IFNB1) was significantly downregulated in other sepsis infection cases compared to its expression in healthy controls (Fig. 1; see also Table S4). IFNA1 and IFNB1 showed upregulation, whereas IFNA5 was downregulated in the melioidosis patients compared to its expression in other sepsis infection cases (Table 2 and Fig. 1; see also Table S2).

Elevated expression of IFN-γ, a proinflammatory cytokine, has been reported in human host and animal models of *B. pseudomallei* infection during the early stages (7, 11, 12, 16). Our findings did not show any significant upregulation of IFN-γ in melioidosis cases compared to its expression in healthy controls or other bacterial sepsis cases, possibly due to our samples being collected at later stages while the patients were undergoing antibiotic treatment. However, we did see significant upregulation in the diabetic melioidosis cohort compared to the expression in other sepsis infection cases (Fig. 2; see also Table S6 in the supplemental material). Interferon-mediated responses have been reported as the most dominant pathway, with class I and II interferons being prominent in melioidosis and tuberculosis infections (28). Our study shows upregulated expression of INFA1 and INFB1 in melioidosis patients (including diabetic and septicemic cohorts) compared to their expression in other sepsis infections (Fig. 1 and 2; see also Table S5 and S6 in the supplemental material). Alpha and beta interferons, both belonging to the class I interferons, play a major role in innate immune responses. Dysregulated type I IFN production results in a damaging cascade of cell death, inflammation, and immunological host responses that can lead to tissue injury and disease progression (29). Studies have shown type I IFN responses to be a striking characteristic of tuberculosis infection and have shown that lack of development of Th1 immunity in response to *Mycobacterium tuberculosis* appears to be associated with increased induction of type 1 IFNs, leading to better bacterial survival and host evasion (30). Furthermore, a study also reported that type 1 IFNs suppress IL-1 production, providing a cellular basis for the anti-inflammatory effects and the probacterial functions of type I IFNs during *M. tuberculosis* infection (31). Our findings show a similar response, as we see a dominant type I IFN production and a fairly submissive production of IFN-γ and related Th1 cytokines in melioidosis patients. These data support our findings of significantly downregulated expression of IL-1A and IL-1B in melioidosis patients compared to their expression in other sepsis cases. Type I interferons are also considered mediators of endotoxic shock and sepsis induced by Gram-negative bacteria, with IFNB and type 1 IFN receptor 1 (IFNAR1) being considered as therapeutic targets (32, 33). Thus, further investigation is required to understand the expression of class I interferons in relation to the pathogenesis of melioidosis and its role in diagnostic and therapeutic intervention.

### Gene expression profile of TNF superfamily.

Our findings reveal an upregulation of TNF-α, an important proinflammatory cytokine, in melioidosis cases compared to its expression in healthy controls (Table 1 and Fig. 1; see also Table S3 in the supplemental material). Several studies have reported the upregulated expression of TNF-α during melioidosis in human host and animal models of infection (7, 11, 12, 34). Elevated plasma concentrations of TNF-α have been correlated with disease severity and mortality in septicemic melioidosis patients (35).

CD40 ligand (CD40L), which plays a major role in B cell activation and development, proinflammatory cytokines, and lymphotoxin alpha (LTA) were downregulated in the melioidosis cohort compared to their expression in healthy controls (Table 1 and Fig. 1; see also Table S3 in the supplemental material). CD40L has been considered an important mediator of sepsis, implicated in the platelet-mediated activation and accumulation of neutrophils during inflammation (36, 37).

Tumor necrosis factor (ligand) superfamily 14 (TNFSF14), which plays a major role in T cell proliferation, TNFSF4, which is responsible for Th2 cell differentiation, and TNFSF8, implicated in blocking Th1 responses, were upregulated in the melioidosis cohort compared to their expression in other sepsis infection cases (Table 1 and Fig. 1; see also Table S2 in the supplemental material). TNFSF4 was consistently upregulated in melioidosis patients compared to its expression in other sepsis cases, irrespective of factors like the duration of clinical symptoms, antibiotic treatment, and comorbidities such as diabetes (Fig. 1 to 3; see also Tables S6 to S8). Upregulated expression of TNFSF4 (also known as OX40L) has been observed in cases of polymicrobial sepsis and autoimmune disease and has been correlated to disease severity and mortality (38). Studies have also shown that upregulated expression of TNFSF4 promoted T cell proliferation and increased the expression of CD4^+^ T cells and the production of Th2 cytokines like IL-4 (39, 40). It has also been postulated as a specific biomarker in therapeutic interventions for treatment of sepsis/septic shock and other autoimmune diseases (38). TNFSF14, otherwise known as LIGHT, plays a major role in the systemic immune response, particularly in long-term survival of memory Th1 and Th2 cells (41). TNFSF8, or CD30L, is reportedly expressed in Th2 cells and suppresses Th1 responses (41). These findings once again suggest an inclination for dominant Th2 responses during the disease progression of melioidosis.

10.1128/mSphere.00121-17.8 TABLE S8  Relative expression ratios of 84 investigated gene targets in PBMCs of melioidosis cases with ≤15 days of antibiotics treatment (*n* = 14) and >15 days of antibiotics treatment (*n* = 8) compared to PBMCs of other sepsis cases (*n* = 10). Shading indicates statistically significant differential gene expression at a *P* value of <0.05. Download TABLE S8, PDF file, 0.2 MB. Copyright © 2017 Krishnananthasivam et al. 2017 Krishnananthasivam et al. This content is distributed under the terms of the Creative Commons Attribution 4.0 International license.

### Gene expression profile of TGF-β superfamily.

Bone morphogenetic protein 6 (BMP6), inhibin beta A (INHBA), and transforming growth factor beta 1 (TGFB1) showed significant upregulation in melioidosis patients compared to their expression in healthy individuals (Table 1 and Fig. 1; see also Table S3 in the supplemental material). BMP3 was downregulated in other sepsis infection cases compared to its expression in healthy controls (Fig. 1; see also Table S4). BMP3, BMP4, BMP6, growth differentiation factor 2 (GDF2), INHBA, and TGFB1 showed upregulated expression in melioidosis patients compared to their expression in other sepsis cases, with BMP4 and GDF2 showing high levels of gene expression (Table 2 and Fig. 1; see also Table S2).

High-level expression of BMP3, BMP6, TGFB1, and TGFB2 was observed in the septicemic melioidosis cohort compared to that in other bacterial sepsis cases (Fig. 1; see also Table S5 in the supplemental material). TGFB2 was expressed at high levels in the early-acute-phase (<15 days of fever/clinical symptoms) melioidosis cohort compared to its levels in sepsis controls (Fig. 3; see also Table S7).

TGF-β was upregulated during melioidosis infection, with increased levels being correlated to severe melioidosis in human hosts (42). Our study revealed a consistent upregulation of TGFB1 in melioidosis patients compared to its expression in other sepsis cases, irrespective of factors like the duration of clinical symptoms, antibiotic treatment, and comorbidities such as diabetes (Fig. 1 to 3; see also Tables S6 to S8). An experimental murine model of melioidosis revealed that the inhibition of TGF-β with a selective TGF-β antibody had a protective effect, with reductions in inflammation, bacterial load, and organ damage, thus indicating the role of TGF-β in the pathogenesis of melioidosis (42). Several other studies have also shown the crucial role of TGF-β in immune regulation, where it induces Foxp3, a master regulator of Tregs in naive T cells, with consequent suppression of proinflammatory cytokines like IFN-γ and enhanced production of anti-inflammatory cytokines (43, 44). It has also been identified as an inducer of Th17 cell differentiation (43, 45). These studies further support our findings of increased Th17 cytokine production and suppression of Th1 cytokines in melioidosis patients.

BMP3 and BMP6 were consistently upregulated in melioidosis patients compared to their expression in other sepsis cases, irrespective of factors like the duration of clinical symptoms, antibiotic treatment, and comorbidities such as diabetes (Fig. 1 to 3; see also Tables S6 to S8). BMPs play a major role in the formation and repair of bone and cartilage and in cell proliferation, differentiation, and apoptosis (46). INHBA overexpression has been associated with increased cell proliferation and poor disease outcome in several types of carcinomas (47, 48). Further studies are needed to elucidate the mechanisms of BMP signaling pathways and INHBA expression in relation to the pathogenesis of melioidosis.

### Gene expression profile of growth factors.

Platelet-derived growth factor alpha (PDGFA) polypeptide was significantly upregulated in melioidosis patients compared to its expression in healthy individuals (Table 1 and Fig. 1; see also Table S3 in the supplemental material). PDGFA, thrombopoietin (THPO), ciliary neurotrophic factor (CNTF), macrophage colony-stimulating factor (M-CSF or CSF1), C-fos-induced growth factor, and vascular endothelial growth factor D (FIGF) showed upregulation in melioidosis cases compared to their expression in other sepsis infection cases (Table 2 and Fig. 1; see also Table S2). Downregulated expression of THPO was observed in other sepsis cases compared to its expression in healthy individuals (Fig. 1; see also Table S4).

PDGF is an important growth factor that plays a crucial role in blood vessel formation (angiogenesis) and regulates cell growth and differentiation. THPO stimulates the production and differentiation of megakaryocytes, thus regulating platelet production. FIGF plays an active role in angiogenesis and vascular endothelial cell growth (49). Increased expression of PDGF is seen in severe bacterial infections, implicating the role of angiogenic factors in endothelial dysfunction leading to disease pathogenesis (49). PDGF has also been suggested as a biomarker of sepsis, related to vascular endothelial damage (50). Our findings also agree with these reports, as we see an increased expression of growth factors, which play a role in endothelial function.

Downregulated expression of leukemia inhibitory factor (LIF), an IL-6 class cytokine that inhibits cell differentiation, and a similar cytokine, oncostatin M (OSM), was observed in the early-acute-phase melioidosis cases (<15 days fever/clinical symptoms) compared to their expression in other sepsis cases (Fig. 3; see also Table S7 in the supplemental material). M-CSF (or CSF1), FIGF, and PDGFA were consistently upregulated in melioidosis patients compared to their expression in other sepsis cases, irrespective of factors like the duration of clinical symptoms, antibiotic treatment, and comorbidities such as diabetes (Fig. 1 to 3; see also Tables S6 to S8). Studies with experimental mouse models of melioidosis have revealed upregulation of mRNA for macrophage colony-stimulating factor (CSF1 or M-CSF), granulocyte macrophage colony-stimulating factor (CSF2 or GM-CSF), and granulocyte colony-stimulating factor (CSF3 or G-CSF) at day 3 postinfection, correlating with peak bacterial load and extensive infiltration of leucocytes (51). Colony-stimulating factors are glycoproteins that are necessary for the survival, proliferation, and differentiation of hematopoietic progenitor cells of the myeloid and erythroid lineage. M-CSF enhances the survival and activation of cells of the monocyte lineage, while GM-CSF and G-CSF increase the accumulation and activation of both neutrophils and macrophages (51). While colony-stimulating factors play a crucial role in innate immune responses and host defense, their high levels of expression during melioidosis may instead contribute to disease pathogenesis.

### Limitations of the study.

The main limitation of our study was that the melioidosis patient samples were collected after the start of antibiotic treatment, which may affect immunocompetent cells, which in turn affects the cytokine profiles studied here. Studies have shown that antibiotics like meropenem exert an immunomodulatory effect, affecting the production of some cytokines in peripheral blood mononuclear cells (PBMCs) (52). This may have been the main reason we could not see any significant differential expression of some key inflammatory response cytokines, such as IFN-γ. The duration of clinical symptoms ranged from >10 days to >90 days, and the duration of antibiotic treatment ranged from 3 days to >30 days at the time of blood collection for all the melioidosis samples. Since our sample collection was nationwide, the duration between patient identification/disease confirmation and sampling was substantial due to logistical issues. Thus, due to the wide range of the duration of the acute phase in the cases whose samples were analyzed and the small number of samples from patients with ≤15 days of fever/clinical symptoms (*n* = 5), we could not see any statistically significant differential expression of some of the inflammatory response genes involved in early innate immune responses. However, our results showed consistently upregulated expression of interleukins IL-4, IL-17A, IL-23A, and IL-24, interferons IFNA1 and IFNB1, TNF superfamily 4 (TNFSF4 or OX40L), TGF superfamily, BMP3, BMP6, TGFB1, and other growth factors, including CSF1, FIGF, and PDGFA, in melioidosis patients compared to their expression in other sepsis cases, irrespective of comorbidities, the duration of fever/clinical symptoms, and antibiotic treatment (see Tables S6 to S8 in the supplemental material), indicating their differential expression during melioidosis infection. Our findings suggest the dominance of Th2- and Th17-type responses during the disease pathogenesis of melioidosis.

As diabetes was seen as a major comorbidity in our experimental cohort, we analyzed our data to see if there was any significant differential expression between diabetic melioidosis cases and nondiabetic melioidosis cases. The gene expression patterns in these two groups were comparable, and we could not find any statistically significant differential expression, indicating that the differential expression was largely due to melioidosis infection (see Table S9 in the supplemental material).

10.1128/mSphere.00121-17.9 TABLE S9  Relative expression ratios of 84 investigated gene targets in PBMCs of diabetic melioidosis cases (*n* = 17) compared to PBMCs of nondiabetic melioidosis cases (*n* = 9). Download TABLE S9, PDF file, 0.1 MB. Copyright © 2017 Krishnananthasivam et al. 2017 Krishnananthasivam et al. This content is distributed under the terms of the Creative Commons Attribution 4.0 International license.

### Conclusion.

Our study revealed differential gene expression of key cytokines involved in human host responses that can distinguish melioidosis cases from sepsis infections caused by other pathogens and healthy individuals. Low levels of expression of key inflammatory mediators IL-1A, IL-1B, and IL-8 were seen in melioidosis patients in early acute phase and with septicemia compared to their expression in other sepsis infection cases. These findings indicate that differentially expressed genes should be validated during different stages of infection for their potential as disease biomarkers for diagnostic purposes and for monitoring disease progression. Our results also show elevated expression of Th17 cytokines like IL-17 and IL-22, as well as TGF-β, which acts as an inducer of Th17 cytokines. Th2 cytokines like IL-3, IL-4, and IL-13 were also upregulated, along with type I interferons and TNFSF cytokines, which are known to be inducers of Th2 cytokines and suppressors of Th1 responses. These results may indicate dominant Th2- and Th17-type cytokine responses, suggesting that their dysregulation may play an important role in disease pathogenesis and progression. IL-17, IL-23, and IL-27, already implicated in therapeutic intervention of several inflammatory diseases, should be further investigated for their role in disease progression and therapeutic approaches in melioidosis.

Our future studies will be aimed at studying gene expression profiles in the early and late acute phases of melioidosis to evaluate candidate genes that could serve as disease and diagnostic biomarkers in different stages of infection. If the antibiotic treatment regime can be adjusted based on these biomarkers, it will bring benefits to patients by reducing their hospital stay. We would expand our studies further, with a larger sample size in each category of sample type, focusing on specific immune response genes showing differential expression to further understand their role in disease susceptibility, pathogenesis, and severity associated with major comorbidities, such as diabetes.

## MATERIALS AND METHODS

### Patient enrollment.

Nationwide active surveillance for melioidosis was established in multiple state and private hospitals throughout Sri Lanka, with ethics approval from the Ethics Review Committee, Faculty of Medicine, University of Colombo, Sri Lanka, and the Office of Human Research Protection (OHRP), United States Army Medical Research and Material Command (USAMRMC). Patients fitting the clinical case definition of melioidosis, i.e., febrile illness for more than 5 days, pneumonia, septic arthritis, skin lesions, septicemia, and lung, soft tissue, or deep abscess, were recruited for initial screening for melioidosis. Blood, pus, and other patient specimens were collected for bacterial cultures, and serum samples were collected for indirect hemagglutination (IHA) antibody testing. Any positive bacterial cultures were further screened and confirmed as *B. pseudomallei* by PCR. All samples for the study were collected between September 2014 and April 2016.

Patients who were culture positive for *B. pseudomallei* and/or had high antibody titers (>640) by the IHA test were recruited for our study and classified as positive cases of melioidosis. Culture- and PCR-positive samples were considered confirmed cases of melioidosis. Samples with an antibody titer of >640 by IHA testing were considered probable cases of melioidosis. At the time of recruitment, all melioidosis patients were undergoing antibacterial treatment.

We also recruited healthy donors and patients fitting the clinical definition of severe sepsis/septic shock (according to the 2012 Surviving Sepsis Campaign guidelines for sepsis management) who were negative for *B. pseudomallei* as negative controls for our gene expression profiling study (53).

### Bacterial culture and identification.

Primary isolation of *B. pseudomallei* was done at the admitting hospital using conventional culture techniques for blood, sputum, pus, and other specimens. Bacterial isolates that were oxidase-positive, gentamicin-resistant, and Gram-negative bacilli were forwarded to the reference laboratory in Colombo, where they were subcultured to establish pure growth and maintained at −70°C in 15% brain heart infusion (BHI)-glycerol for subsequent definitive tests. Bacteria were resuscitated by subculture onto 5% blood agar and incubated for 24 h at 37°C to give single-colony growth for all subsequent tests.

### Real-time PCR assay for confirmation of *B. pseudomallei.*

A single colony of *B. pseudomallei* grown on blood agar from a patient’s sample was resuspended in ultrapure water. The suspension was heated at 95°C for 10 min and centrifuged at 13,500 × *g* to pellet the cell debris. The supernatant was used as the template for all subsequent PCR assays. A real-time PCR assay was done for gene targets of the *lpxO*, *YLF*, and *BTFC* gene clusters using primers and methods described previously (54, 55).

### IHA antibody testing.

Antibody testing against *B. pseudomallei* antigen was performed using an in-house method adapted from Alexander et al. (56). Antigen was prepared from heat-killed culture supernatant of the Sri Lankan *B. pseudomallei* strain BPs7. An antigen preparation at 1/80 dilution was used to sensitize sheep erythrocytes. Serum samples were heat inactivated at 56°C for 30 min and tested by serial dilution from 1/10 to 1/10,240 with sensitized sheep erythrocytes, and the reciprocal of the highest dilution at which hemagglutination occurred was recorded as the endpoint titer (56).

### Sample collection and processing

Ten milliliters of whole blood was collected from patients/volunteers after written informed consent, of which 7 ml was collected into BD Vacutainer mononuclear cell preparation tubes (catalog number 362761) for lymphocyte purification. The lymphocytes were purified using the Ficoll fractionation method according to the manufacturer’s instructions, lysed with RLT buffer (RNeasy minikit, catalog number 74104; Qiagen), homogenized, and stored at −80°C for total RNA extraction.

### Total RNA extraction and cDNA synthesis

Total RNA was extracted from the stored cell lysate samples using the Qiagen RNeasy minikit (catalog number 74104) according to the manufacturer’s protocol. RNA extracted from 0.6 million PBMCs was used for cDNA synthesis as the standard for all samples analyzed by real-time quantitative PCR (RT-qPCR). cDNA was synthesized using the Qiagen first-strand kit (catalog number 330401) according to the manufacturer’s recommendations. The synthesized cDNA samples were stored at −20°C until further use.

### Real-time qPCR and gene expression analysis.

The Qiagen human common cytokines RT^2^ Profiler PCR array (catalog number PAHS-021Z) was used for this study. The PCR protocol and thermal profile recommended by the manufacturer were followed. PBMCs from 26 melioidosis cases (identified as confirmed or probable cases), 10 other bacterial sepsis cases (negative for *B. pseudomallei*), and 5 healthy negative controls were analyzed by RT-qPCR.

### Data analysis

The relative gene expression ratio for measuring the change in the expression level of a gene was calculated by the cycle threshold (ΔΔ*C*_*T*_) method (57) according to the manufacturer’s recommendations. The data were normalized using beta actin as the reference housekeeping gene. Statistical analysis was done using Welch’s *t* test in SAS PROC MIXED, version 9.4. A *P* value of <0.05 was considered statistically significant.
