# Supplementary material for: Gene Expression Profile of Human Cytokines in Response to Burkholderia pseudomallei Infection
Source: mSphere. 2017 Apr 19;2(2):e00121-17. doi: 10.1128/mSphere.00121-17 (PMC5397567; doi:10.1128/mSphere.00121-17)
Supplement: TABLE S1 [file sph002172268st1.pdf]

Table S1

Gene Target Symbol	Full Name of Gene target
ADIPOQ	Adiponectin, C1Q and collagen domain containing
BMP1	Bone morphogenetic protein 1
BMP2	Bone morphogenetic protein 2
BMP3	Bone morphogenetic protein 3
BMP4	Bone morphogenetic protein 4
BMP5	Bone morphogenetic protein 5
BMP6	Bone morphogenetic protein 6
BMP7	Bone morphogenetic protein 7
CD40LG	CD40 ligand
CD70	CD70 molecule
CNTF	Ciliary neurotrophic factor
CSF1	Colony stimulating factor 1 (macrophage)
CSF2	Colony stimulating factor 2 (granulocyte-macrophage)
CSF3	Colony stimulating factor 3 (granulocyte)
FAM3B	Family with sequence similarity 3, member B
FASLG	Fas ligand (TNF superfamily, member 6)
FIGF	C-fos induced growth factor (vascular endothelial growth factor D)
GDF2	Growth differentiation factor 2
GDF5	Growth differentiation factor 5
GDF9	Growth differentiation factor 9
IFNA1	Interferon, alpha 1
IFNA2	Interferon, alpha 2
IFNA4	Interferon, alpha 4
IFNA5	Interferon, alpha 5
IFNB1	Interferon, beta 1, fibroblast
IFNG	Interferon, gamma
IL10	Interleukin 10
IL11	Interleukin 11
IL12A	Interleukin 12A (cytotoxic lymphocyte maturation factor 1, p35)
IL12B	Interleukin 12B (cytotoxic lymphocyte maturation factor 2, p40)
IL13	Interleukin 13
IL15	Interleukin 15
IL16	Interleukin 16
IL17A	Interleukin 17A
IL17B	Interleukin 17B
IL17C	Interleukin 17C
IL18	Interleukin 18 (interferon-gamma-inducing factor)
IL19	Interleukin 19
IL1A	Interleukin 1, alpha
IL1B	Interleukin 1, beta
IL1RN	Interleukin 1 receptor antagonist

IL2	Interleukin 2
IL20	Interleukin 20
IL21	Interleukin 21
IL22	Interleukin 22
IL23A	Interleukin 23, alpha subunit p19
IL24	Interleukin 24
IL25	Interleukin 25
IL27	Interleukin 27
IL3	Interleukin 3 (colony-stimulating factor, multiple)
IL4	Interleukin 4
IL5	Interleukin 5 (colony-stimulating factor, eosinophil)
IL6	Interleukin 6 (interferon, beta 2)
IL7	Interleukin 7
IL8	Interleukin 8
IL9	Interleukin 9
INHA	Inhibin, alpha
INHBA	Inhibin, beta A
LEFTY2	Left-right determination factor 2
LIF	Leukemia inhibitory factor (cholinergic differentiation factor)
LTA	Lymphotoxin alpha (TNF superfamily, member 1)
LTB	Lymphotoxin beta (TNF superfamily, member 3)
MSTN	Myostatin
NODAL	Nodal homolog (mouse)
OSM	Oncostatin M
PDGFA	Platelet-derived growth factor alpha polypeptide
SPP1	Secreted phosphoprotein 1
TGFA	Transforming growth factor, alpha
TGFB1	Transforming growth factor, beta 1
TGFB2	Transforming growth factor, beta 2
TGFB3	Transforming growth factor, beta 3
THPO	Thrombopoietin
TNF	Tumor necrosis factor
TNFRSF11B	Tumor necrosis factor receptor superfamily, member 11b
TNFSF10	Tumor necrosis factor (ligand) superfamily, member 10
TNFSF11	Tumor necrosis factor (ligand) superfamily, member 11
TNFSF12	Tumor necrosis factor (ligand) superfamily, member 12
TNFSF13	Tumor necrosis factor (ligand) superfamily, member 13
TNFSF13B	Tumor necrosis factor (ligand) superfamily, member 13B
TNFSF14	Tumor necrosis factor (ligand) superfamily, member 14
TNFSF4	Tumor necrosis factor (ligand) superfamily, member 4
TNFSF8	Tumor necrosis factor (ligand) superfamily, member 8
TXLNA	Taxilin alpha
VEGFA	Vascular endothelial growth factor A
