# Supplementary material for: Gene Expression Profile of Human Cytokines in Response to Burkholderia pseudomallei Infection
Source: mSphere. 2017 Apr 19;2(2):e00121-17. doi: 10.1128/mSphere.00121-17 (PMC5397567; doi:10.1128/mSphere.00121-17)
Supplement: TABLE S2 [file sph002172268st2.pdf]

Table S2

Gene Target	Relative Expression Ratio	Confidence Limit	P-Value
ADIPOQ	1.993	0.273 , 14.521	0.4712
BMP1	1.243	0.851 , 1.814	0.2432
BMP2	2.329	0.901 , 6.018	0.0788
BMP3	5.305	2.319 , 12.135	0.0003
BMP4	18.765	1.479 , 238.054	0.0271
BMP5	3.423	0.594 , 19.741	0.1586
BMP6	2.776	1.214 , 6.344	0.0192
BMP7	1.346	0.116 , 15.615	0.8062
CD40LG	2.275	0.991 , 5.222	0.0521
CD70	1.667	0.856 , 3.245	0.119
CNTF	2.222	1.023 , 4.829	0.0441
CSF1	2.456	1.451 , 4.156	0.0017
CSF2	2.331	0.602 , 9.033	0.2049
CSF3	2.178	0.981 , 4.834	0.0554
FAM3B	3.195	0.692 , 14.766	0.1278
FASLG	1.939	0.648 , 5.806	0.213
FIGF	3.912	1.561 , 9.802	0.0049
GDF2	11.112	1.105 , 111.704	0.0421
GDF5	2.957	0.762 , 11.476	0.1116
GDF9	1.258	0.541 , 2.924	0.5815
IFNA1	4.034	1.358 , 11.984	0.014
IFNA2	17.777	0.046 , +Inf	0.2395
IFNA4	5.844	0.894 , 38.217	0.0635
IFNA5	0.2	0.057 , 0.704	0.0152
IFNB1	3.206	1.056 , 9.735	0.0407
IFNG	2.471	0.907 , 6.736	0.0742
IL10	0.842	0.364 , 1.949	0.6761
IL11	1.217	0.446 , 3.321	0.6885
IL12A	1.549	0.644 , 3.725	0.2994
IL12B	2.497	0.874 , 7.133	0.0846
IL13	2.529	0.896 , 7.142	0.0767
IL15	1.271	0.925 , 1.746	0.1335
IL16	1.896	1.152 , 3.121	0.0157
IL17A	16.32	3.193 , 83.421	0.0017
IL17B	2.939	1.486 , 5.811	0.003
IL17C	1.623	0.793 , 3.322	0.1783
IL18	0.906	0.572 , 1.435	0.6537
IL19	1.712	0.700 , 4.188	0.2267
IL1A	0.39	0.114 , 1.328	0.1255
IL1B	0.615	0.208 , 1.817	0.363

IL1RN	1.747	1.091 , 2.796	0.0216
IL2	1.387	0.458 , 4.204	0.5363
IL20	2.793	0.592 , 13.168	0.1781
IL21	0.723	0.283 , 1.845	0.4849
IL22	3.022	1.207 , 7.565	0.0206
IL23A	2.792	1.329 , 5.866	0.0092
IL24	2.991	1.240 , 7.214	0.0173
IL25	2.227	0.587 , 8.454	0.2255
IL27	3.089	1.203 , 7.932	0.0206
IL3	9.38	1.773 , 49.626	0.0107
IL4	6.024	1.153 , 31.479	0.0344
IL5	1.105	0.445 , 2.740	0.8222
IL6	1.503	0.529 , 4.269	0.4203
IL7	1.473	0.819 , 2.650	0.1862
IL8	0.567	0.225 , 1.432	0.2208
IL9	2.968	0.350 , 25.181	0.2747
INHA	5.481	0.616 , 48.808	0.1188
INHBA	4.635	1.205 , 17.822	0.0282
LEFTY2	1.606	0.650 , 3.971	0.2851
LIF	1.516	0.509 , 4.513	0.4405
LTA	1.53	0.751 , 3.116	0.2289
LTB	1.841	0.905 , 3.744	0.0865
MSTN	2.135	0.502 , 9.086	0.2864
NODAL	1.275	0.702 , 2.317	0.403
OSM	1.668	0.791 , 3.517	0.1713
PDGFA	2.317	1.065 , 5.038	0.0357
SPP1	1.832	0.414 , 8.097	0.4081
TGFA	1.18	0.595 , 2.341	0.6225
TGFB1	2.006	1.374 , 2.931	0.0007
TGFB2	3.674	0.767 , 17.593	0.0954
TGFB3	1.82	0.840 , 3.942	0.1205
THPO	4.213	1.042 , 17.040	0.0441
TNF	0.91	0.260 , 3.187	0.8744
TNFRSF11B	0.956	0.186 , 4.901	0.9542
TNFSF10	1.405	0.876 , 2.252	0.1513
TNFSF11	1.49	0.561 , 3.956	0.4055
TNFSF12	1.211	0.799 , 1.836	0.3549
TNFSF13	1.275	0.862 , 1.886	0.2144
TNFSF13B	1.38	0.924 , 2.062	0.1114
TNFSF14	2.353	1.171 , 4.728	0.0186
TNFSF4	2.349	1.167 , 4.728	0.0202
TNFSF8	1.606	1.004 , 2.571	0.0484
TXLNA	1.216	0.817 , 1.809	0.3136
VEGFA	1.22	0.558 , 2.671	0.5991
