# Supplementary material for: Gene Expression Profile of Human Cytokines in Response to Burkholderia pseudomallei Infection
Source: mSphere. 2017 Apr 19;2(2):e00121-17. doi: 10.1128/mSphere.00121-17 (PMC5397567; doi:10.1128/mSphere.00121-17)
Supplement: TABLE S3 [file sph002172268st3.pdf]

Table S3

Gene Target	Relative Expression Ratio	Confidence Limit	P-Value
ADIPOQ	0.253	0.064 , 1.002	0.0503
BMP1	1.231	0.748 , 2.027	0.3544
BMP2	0.465	0.003 , 82.471	0.7084
BMP3	0.733	0.188 , 2.852	0.6055
BMP4	1.462	0.078 , 27.489	0.7375
BMP5	1.196	0.073 , 19.576	0.883
BMP6	2.946	1.470 , 5.904	0.007
BMP7	0.026	<0.001 , 1.719	0.0774
CD40LG	0.502	0.336 , 0.752	0.002
CD70	1.024	0.462 , 2.269	0.9409
CNTF	1.638	0.712 , 3.771	0.235
CSF1	1.447	0.806 , 2.597	0.1923
CSF2	1.342	0.573 , 3.140	0.4826
CSF3	1.725	0.677 , 4.399	0.238
FAM3B	0.479	0.151 , 1.520	0.1972
FASLG	0.836	0.381 , 1.835	0.6134
FIGF	2.567	0.667 , 9.871	0.1475
GDF2	40.68	0.484 , +Inf	0.0723
GDF5	2.33	0.236 , 23.003	0.4095
GDF9	0.759	0.379 , 1.520	0.4227
IFNA1	4.293	0.004 , +Inf	0.4904
IFNA2	60.488	0.152 , +Inf	0.1705
IFNA4	2.14	0.189 , 24.199	0.4548
IFNA5	0.189	0.037 , 0.961	0.0454
IFNB1	0.846	0.298 , 2.400	0.7333
IFNG	1.437	0.670 , 3.082	0.3288
IL10	2.257	1.180 , 4.319	0.0158
IL11	0.411	0.061 , 2.756	0.2777
IL12A	0.944	0.618 , 1.442	0.7774
IL12B	5.862	0.943 , 36.417	0.0557
IL13	1.976	0.727 , 5.374	0.1595
IL15	1.28	0.781 , 2.096	0.2806
IL16	0.683	0.527 , 0.887	0.0062
IL17A	2.382	0.433 , 13.100	0.2995
IL17B	1.514	0.584 , 3.927	0.3515
IL17C	1.072	0.559 , 2.058	0.8283
IL18	0.921	0.690 , 1.229	0.5573
IL19	1.241	0.198 , 7.785	0.7664
IL1A	0.911	0.025 , 33.193	0.9376
IL1B	2.504	1.229 , 5.100	0.0135
IL1RN	1.62	1.023 , 2.564	0.0403

IL2	0.613	0.266 , 1.411	0.2255
IL20	0.916	0.290 , 2.891	0.8705
IL21	2.009	0.609 , 6.630	0.2248
IL22	2.75	0.801 , 9.437	0.094
IL23A	1.074	0.648 , 1.780	0.7724
IL24	0.855	0.472 , 1.549	0.5938
IL25	0.988	0.136 , 7.207	0.9891
IL27	4.022	1.632 , 9.915	0.0039
IL3	3.533	0.149 , 83.708	0.3642
IL4	1.941	0.251 , 15.033	0.5041
IL5	1.009	0.364 , 2.792	0.9852
IL6	1.844	0.951 , 3.574	0.0682
IL7	0.852	0.473 , 1.537	0.5777
IL8	2.953	1.394 , 6.257	0.0062
IL9	0.91	<0.001 , +Inf	0.9909
INHA	3.104	0.877 , 10.987	0.0747
INHBA	6.07	2.652 , 13.891	0.0002
LEFTY2	1.444	0.630 , 3.308	0.3485
LIF	1.099	0.464 , 2.605	0.8228
LTA	0.327	0.190 , 0.565	0.0003
LTB	0.517	0.343 , 0.778	0.0031
MSTN	0.545	0.166 , 1.792	0.2938
NODAL	0.703	0.380 , 1.300	0.2252
OSM	1.207	0.609 , 2.390	0.5742
PDGFA	2.86	1.444 , 5.667	0.0066
SPP1	2.004	0.693 , 5.795	0.1902
TGFA	1.792	0.197 , 16.297	0.6007
TGFB1	1.634	1.121 , 2.383	0.0126
TGFB2	1.723	0.766 , 3.876	0.1713
TGFB3	1.117	0.717 , 1.741	0.6126
THPO	0.701	0.134 , 3.658	0.6465
TNF	2.248	1.082 , 4.670	0.0315
TNFRSF11B	0.671	<0.001 , +Inf	0.8718
TNFSF10	1.143	0.720 , 1.816	0.5492
TNFSF11	1.003	0.058 , 17.428	0.9981
TNFSF12	0.865	0.604 , 1.238	0.4144
TNFSF13	1.137	0.628 , 2.058	0.6216
TNFSF13B	1.097	0.725 , 1.658	0.6461
TNFSF14	1.086	0.554 , 2.129	0.7947
TNFSF4	1.497	0.638 , 3.513	0.2914
TNFSF8	1.039	0.759 , 1.421	0.8012
TXLNA	0.862	0.660 , 1.126	0.2604
VEGFA	1.218	0.113 , 13.076	0.8692
