# Supplementary material for: Gene Expression Profile of Human Cytokines in Response to Burkholderia pseudomallei Infection
Source: mSphere. 2017 Apr 19;2(2):e00121-17. doi: 10.1128/mSphere.00121-17 (PMC5397567; doi:10.1128/mSphere.00121-17)
Supplement: TABLE S4 [file sph002172268st4.pdf]

Table S4

Gene Target	Relative Expression Ratio	Confidence Limit	P-Value
ADIPOQ	0.127	0.021 , 0.773	0.0295
BMP1	0.991	0.583 , 1.684	0.9702
BMP2	0.2	0.001 , 35.999	0.443
BMP3	0.138	0.035 , 0.542	0.0113
BMP4	0.078	0.004 , 1.730	0.0853
BMP5	0.349	0.021 , 5.904	0.408
BMP6	1.061	0.422 , 2.670	0.891
BMP7	0.019	<0.001 , 1.317	0.0609
CD40LG	0.221	0.097 , 0.501	0.0019
CD70	0.614	0.249 , 1.516	0.2601
CNTF	0.737	0.425 , 1.280	0.2377
CSF1	0.589	0.321 , 1.082	0.0813
CSF2	0.576	0.161 , 2.055	0.3616
CSF3	0.792	0.371 , 1.691	0.492
FAM3B	0.15	0.034 , 0.656	0.0162
FASLG	0.431	0.132 , 1.406	0.1481
FIGF	0.656	0.174 , 2.475	0.4768
GDF2	3.661	0.050 , 267.877	0.3801
GDF5	0.788	0.080 , 7.810	0.8102
GDF9	0.603	0.312 , 1.167	0.1215
IFNA1	1.064	<0.001 , +Inf	0.9748
IFNA2	3.403	0.004 , +Inf	0.7074
IFNA4	0.366	0.031 , 4.351	0.353
IFNA5	0.945	0.238 , 3.755	0.9212
IFNB1	0.264	0.084 , 0.824	0.0261
IFNG	0.582	0.215 , 1.576	0.261
IL10	2.681	1.294 , 5.557	0.012
IL11	0.338	0.050 , 2.274	0.2027
IL12A	0.609	0.256 , 1.449	0.2338
IL12B	2.347	0.372 , 14.797	0.3055
IL13	0.781	0.265 , 2.308	0.6084
IL15	1.007	0.614 , 1.650	0.9744
IL16	0.36	0.222 , 0.586	0.0007
IL17A	0.146	0.037 , 0.570	0.0104
IL17B	0.515	0.200 , 1.325	0.1423
IL17C	0.661	0.381 , 1.146	0.1278
IL18	1.017	0.646 , 1.600	0.9376
IL19	0.724	0.087 , 6.042	0.6297
IL1A	2.336	0.058 , 93.760	0.4949
IL1B	4.07	1.657 , 9.995	0.0062

IL1RN	0.927	0.645 , 1.333	0.6585
IL2	0.442	0.140 , 1.392	0.1467
IL20	0.328	0.065 , 1.647	0.1594
IL21	2.778	0.867 , 8.897	0.0777
IL22	0.91	0.250 , 3.310	0.8721
IL23A	0.385	0.194 , 0.762	0.0102
IL24	0.286	0.130 , 0.632	0.005
IL25	0.444	0.058 , 3.415	0.3844
IL27	1.302	0.595 , 2.851	0.4779
IL3	0.377	0.015 , 9.614	0.4467
IL4	0.322	0.060 , 1.733	0.1494
IL5	0.913	0.305 , 2.732	0.858
IL6	1.227	0.448 , 3.361	0.6661
IL7	0.579	0.333 , 1.006	0.0522
IL8	5.205	2.553 , 10.613	0.0003
IL9	0.306	<0.001 , +Inf	0.8883
INHA	0.566	0.081 , 3.941	0.5243
INHBA	1.309	0.353 , 4.851	0.6613
LEFTY2	0.899	0.331 , 2.439	0.8204
LIF	0.725	0.325 , 1.616	0.3937
LTA	0.214	0.113 , 0.404	0.0002
LTB	0.281	0.140 , 0.561	0.0019
MSTN	0.255	0.059 , 1.107	0.0656
NODAL	0.551	0.272 , 1.116	0.0906
OSM	0.723	0.368 , 1.424	0.3199
PDGFA	1.235	0.524 , 2.908	0.6036
SPP1	1.094	0.306 , 3.917	0.8796
TGFA	1.518	0.169 , 13.614	0.7061
TGFB1	0.815	0.615 , 1.078	0.1363
TGFB2	0.469	0.097 , 2.267	0.3143
TGFB3	0.614	0.304 , 1.241	0.1539
THPO	0.166	0.033 , 0.838	0.0333
TNF	2.471	0.738 , 8.273	0.1282
TNFRSF11B	0.702	<0.001 , +Inf	0.8862
TNFSF10	0.814	0.507 , 1.306	0.3611
TNFSF11	0.674	0.038 , 11.925	0.7856
TNFSF12	0.714	0.519 , 0.982	0.04
TNFSF13	0.892	0.492 , 1.617	0.6487
TNFSF13B	0.794	0.545 , 1.158	0.2055
TNFSF14	0.462	0.219 , 0.974	0.0436
TNFSF4	0.637	0.247 , 1.643	0.3155
TNFSF8	0.647	0.406 , 1.029	0.0637
TXLNA	0.709	0.484 , 1.039	0.0736
VEGFA	0.998	0.092 , 10.780	0.9987
