# Supplementary material for: Gene Expression Profile of Human Cytokines in Response to Burkholderia pseudomallei Infection
Source: mSphere. 2017 Apr 19;2(2):e00121-17. doi: 10.1128/mSphere.00121-17 (PMC5397567; doi:10.1128/mSphere.00121-17)
Supplement: TABLE S5 [file sph002172268st5.pdf]

Table S5

Gene Target	Relative Expression Ratio	Confidence Limit	P-Value
ADIPOQ	1.521	0.138 , 16.728	0.7142
BMP1	1.092	0.727 , 1.641	0.6561
BMP2	1.361	0.476 , 3.887	0.5464
BMP3	5.335	1.949 , 14.604	0.0022
BMP4	6.364	0.630 , 64.275	0.1012
BMP5	3.918	0.474 , 32.358	0.1885
BMP6	2.913	1.261 , 6.728	0.0159
BMP7	0.392	0.040 , 3.818	0.4009
CD40LG	2.283	0.973 , 5.356	0.0568
CD70	1.623	0.813 , 3.240	0.154
CNTF	1.89	0.626 , 5.710	0.2411
CSF1	1.772	1.008 , 3.115	0.0473
CSF2	1.966	0.466 , 8.284	0.3381
CSF3	1.57	0.513 , 4.804	0.4085
FAM3B	2.73	0.568 , 13.129	0.1944
FASLG	1.54	0.503 , 4.715	0.4207
FIGF	3.16	1.066 , 9.370	0.0389
GDF2	6.62	0.420 , 104.364	0.1547
GDF5	1.768	0.435 , 7.192	0.4032
GDF9	1.647	0.810 , 3.346	0.1565
IFNA1	3.559	1.272 , 9.961	0.0181
IFNA2	7.189	0.016 , +Inf	0.3951
IFNA4	5.894	0.862 , 40.304	0.0674
IFNA5	0.175	0.037 , 0.832	0.0318
IFNB1	4.314	1.088 , 17.096	0.0385
IFNG	2.019	0.668 , 6.099	0.2007
IL10	0.497	0.197 , 1.253	0.1316
IL11	0.942	0.268 , 3.308	0.9209
IL12A	1.211	0.505 , 2.900	0.6421
IL12B	2.187	0.707 , 6.764	0.1654
IL13	2.601	0.822 , 8.232	0.0986
IL15	1.08	0.780 , 1.495	0.6312
IL16	1.589	0.959 , 2.633	0.0691
IL17A	11.695	1.845 , 74.130	0.0122
IL17B	2.469	1.064 , 5.731	0.0365
IL17C	1.19	0.600 , 2.358	0.6048
IL18	0.834	0.527 , 1.318	0.4089
IL19	0.841	0.291 , 2.431	0.7308
IL1A	0.199	0.055 , 0.723	0.0171
IL1B	0.264	0.095 , 0.731	0.0134

IL1RN	1.262	0.735 , 2.167	0.3819
IL2	1.075	0.312 , 3.705	0.9033
IL20	2.325	0.423 , 12.792	0.3132
IL21	0.791	0.259 , 2.416	0.6674
IL22	2.753	0.924 , 8.200	0.0676
IL23A	2.232	1.042 , 4.780	0.0399
IL24	2.287	0.973 , 5.372	0.0569
IL25	1.381	0.283 , 6.740	0.6755
IL27	2.646	0.754 , 9.287	0.1221
IL3	11.644	0.949 , 142.938	0.0542
IL4	6.092	0.401 , 92.675	0.1755
IL5	0.982	0.356 , 2.707	0.9699
IL6	1.079	0.344 , 3.382	0.8907
IL7	1.238	0.578 , 2.652	0.5652
IL8	0.317	0.114 , 0.881	0.0292
IL9	3.264	0.384 , 27.716	0.2382
INHA	2.083	0.161 , 27.006	0.5499
INHBA	2.439	0.674 , 8.828	0.1563
LEFTY2	1.261	0.502 , 3.163	0.6021
LIF	0.776	0.217 , 2.776	0.6813
LTA	1.226	0.507 , 2.965	0.6378
LTB	1.641	0.788 , 3.414	0.1712
MSTN	1.534	0.305 , 7.731	0.5881
NODAL	1.031	0.557 , 1.908	0.9187
OSM	0.963	0.455 , 2.040	0.9182
PDGFA	2.433	1.059 , 5.593	0.0375
SPP1	1.061	0.216 , 5.220	0.9393
TGFA	0.881	0.396 , 1.957	0.7426
TGFB1	1.661	1.247 , 2.212	0.0013
TGFB2	5.201	1.088 , 24.857	0.0405
TGFB3	1.579	0.673 , 3.701	0.2777
THPO	1.866	0.329 , 10.570	0.4536
TNF	0.514	0.152 , 1.737	0.2584
TNFRSF11B	1.688	0.284 , 10.023	0.5406
TNFSF10	1.285	0.779 , 2.120	0.3117
TNFSF11	1.388	0.522 , 3.686	0.4908
TNFSF12	1.067	0.615 , 1.852	0.8096
TNFSF13	1.092	0.781 , 1.526	0.5896
TNFSF13B	1.06	0.725 , 1.550	0.7543
TNFSF14	1.63	0.770 , 3.447	0.1902
TNFSF4	2.282	1.096 , 4.752	0.0298
TNFSF8	1.401	0.870 , 2.255	0.1524
TXLNA	1.06	0.707 , 1.588	0.7664
VEGFA	0.894	0.367 , 2.180	0.7952
