# Supplementary material for: Gene Expression Profile of Human Cytokines in Response to Burkholderia pseudomallei Infection
Source: mSphere. 2017 Apr 19;2(2):e00121-17. doi: 10.1128/mSphere.00121-17 (PMC5397567; doi:10.1128/mSphere.00121-17)
Supplement: TABLE S6 [file sph002172268st6.pdf]

Table S6

	diabetic melioidosis vs sepsis			diabetic melioidosis vs healthy		
**Gene Target**	**Relative Expression Ratio**	**Confidence Limit**	**P-Value**	**Relative Expression Ratio**	**Confidence Limit**	**P-Value**
ADIPOQ	3.116	0.406 , 23.930	0.2532	0.396	0.090 , 1.736	0.1988
BMP1	1.243	0.827 , 1.870	0.2799	1.232	0.739 , 2.054	0.3765
BMP2	2.52	0.777 , 8.178	0.1171	0.503	0.003 , 86.911	0.7395
BMP3	5.953	2.228 , 15.902	0.001	0.823	0.200 , 3.380	0.763
BMP4	14.115	0.777 , 256.374	0.0699	1.1	0.050 , 24.107	0.9412
BMP5	2.586	0.429 , 15.604	0.2833	0.904	0.055 , 14.968	0.9345
BMP6	2.756	1.141 , 6.660	0.0267	2.925	1.378 , 6.211	0.0091
BMP7	1.996	0.119 , 33.605	0.6164	0.038	<0.001 , 2.811	0.117
CD40LG	2.382	1.005 , 5.644	0.0489	0.526	0.324 , 0.855	0.0122
CD70	1.743	0.895 , 3.395	0.0935	1.071	0.483 , 2.372	0.8315
CNTF	2.878	0.981 , 8.445	0.0538	2.122	0.698 , 6.453	0.1734
CSF1	2.97	1.621 , 5.440	0.0011	1.75	0.915 , 3.347	0.0855
CSF2	3.331	0.769 , 14.433	0.1026	1.917	0.671 , 5.478	0.2108
CSF3	2.766	1.050 , 7.286	0.0404	2.191	0.749 , 6.412	0.1427
FAM3B	7.344	1.571 , 34.329	0.0144	1.101	0.339 , 3.574	0.8648
FASLG	2.082	0.677 , 6.407	0.1828	0.898	0.395 , 2.039	0.7756
FIGF	4.41	1.456 , 13.362	0.0108	2.894	0.683 , 12.268	0.1349
GDF2	10.905	1.292 , 92.069	0.0318	39.919	0.163 , +Inf	0.0953

GDF5	2.133	0.605 , 7.512	0.2225	1.681	0.172 , 16.387	0.5958
GDF9	1.177	0.379 , 3.650	0.7678	0.71	0.251 , 2.011	0.4957
IFNA1	3.726	1.066 , 13.016	0.0401	3.965	0.004 , +Inf	0.5133
IFNA2	7.982	0.016 , +Inf	0.3624	27.159	0.080 , +Inf	0.2541
IFNA4	4.38	0.457 , 42.015	0.1803	1.604	0.118 , 21.807	0.6775
IFNA5	0.2	0.037 , 1.084	0.06	0.189	0.028 , 1.302	0.0849
IFNB1	4.432	1.116 , 17.594	0.0355	1.169	0.313 , 4.368	0.8051
IFNG	3.282	1.100 , 9.788	0.0344	1.909	0.786 , 4.633	0.1433
IL10	1.307	0.542 , 3.148	0.5356	3.504	1.726 , 7.110	0.0014
IL11	1.371	0.471 , 3.993	0.5458	0.463	0.069 , 3.089	0.3474
IL12A	1.566	0.643 , 3.811	0.2961	0.954	0.603 , 1.510	0.8313
IL12B	2.89	0.869 , 9.614	0.0809	6.784	1.051 , 43.809	0.0455
IL13	3.716	1.102 , 12.526	0.0357	2.904	0.899 , 9.375	0.0711
IL15	1.366	0.956 , 1.953	0.0841	1.376	0.827 , 2.290	0.1909
IL16	1.858	1.104 , 3.125	0.0226	0.67	0.490 , 0.915	0.0144
IL17A	14.8	2.526 , 86.706	0.0049	2.16	0.346 , 13.482	0.3858
IL17B	3.363	1.736 , 6.513	0.0009	1.732	0.673 , 4.459	0.218
IL17C	1.908	0.835 , 4.358	0.1196	1.261	0.581 , 2.735	0.5399
IL18	0.892	0.552 , 1.441	0.6213	0.907	0.654 , 1.257	0.537
IL19	1.989	0.641 , 6.174	0.2168	1.441	0.229 , 9.078	0.6437
IL1A	0.338	0.074 , 1.550	0.1529	0.791	0.027 , 22.920	0.8518
IL1B	0.817	0.251 , 2.660	0.7271	3.326	1.384 , 7.995	0.0102

IL1RN	1.882	1.031 , 3.434	0.0403	1.745	0.964 , 3.157	0.0643
IL2	1.361	0.395 , 4.693	0.6072	0.602	0.219 , 1.656	0.3031
IL20	2.602	0.455 , 14.870	0.2653	0.854	0.207 , 3.517	0.815
IL21	0.79	0.227 , 2.747	0.6981	2.195	0.534 , 9.023	0.2553
IL22	4.123	1.539 , 11.045	0.0069	3.752	1.061 , 13.265	0.042
IL23A	3.501	1.523 , 8.047	0.0049	1.347	0.706 , 2.571	0.348
IL24	3.692	1.521 , 8.959	0.0061	1.056	0.574 , 1.941	0.8542
IL25	2.847	0.720 , 11.248	0.128	1.263	0.171 , 9.327	0.7905
IL27	3.623	1.081 , 12.150	0.038	4.719	1.451 , 15.344	0.0125
IL3	3.761	0.796 , 17.779	0.0896	1.416	0.059 , 33.877	0.7898
IL4	10.845	1.100 , 106.869	0.0421	3.493	0.275 , 44.409	0.3148
IL5	1.598	0.616 , 4.149	0.3183	1.459	0.510 , 4.175	0.4431
IL6	2.274	0.761 , 6.794	0.1325	2.789	1.311 , 5.936	0.0104
IL7	1.851	0.869 , 3.942	0.1046	1.071	0.503 , 2.281	0.8504
IL8	0.93	0.337 , 2.566	0.884	4.841	2.030 , 11.541	0.0012
IL9	2.111	0.234 , 19.026	0.4643	0.647	<0.001 , +Inf	0.9584
INHA	6.072	0.627 , 58.768	0.1121	3.438	0.801 , 14.751	0.0883
INHBA	5.273	1.322 , 21.028	0.0214	6.905	2.802 , 17.014	0.0003
LEFTY2	1.904	0.730 , 4.969	0.176	1.712	0.707 , 4.142	0.2104
LIF	1.694	0.479 , 5.994	0.3966	1.228	0.412 , 3.659	0.6944
LTA	1.648	0.695 , 3.910	0.2446	0.353	0.167 , 0.744	0.0087

LTB	1.9	0.915 , 3.944	0.0809	0.533	0.339 , 0.840	0.0092
MSTN	2.272	0.432 , 11.949	0.3166	0.58	0.136 , 2.474	0.4396
NODAL	1.539	0.805 , 2.940	0.1803	0.848	0.440 , 1.633	0.5905
OSM	2.241	0.987 , 5.085	0.0534	1.621	0.754 , 3.483	0.2023
PDGFA	2.483	1.066 , 5.786	0.0364	3.065	1.439 , 6.530	0.0068
SPP1	1.78	0.282 , 11.239	0.5237	1.948	0.408 , 9.310	0.3799
TGFA	1.775	0.780 , 4.039	0.1599	2.694	0.285 , 25.451	0.3826
TGFB1	2.103	1.369 , 3.231	0.0016	1.713	1.117 , 2.627	0.0162
TGFB2	4.582	0.902 , 23.265	0.0643	2.149	0.833 , 5.547	0.1068
TGFB3	1.983	0.858 , 4.580	0.1038	1.217	0.687 , 2.155	0.4799
THPO	6.014	1.503 , 24.063	0.0137	1.001	0.193 , 5.193	0.9991
TNF	0.891	0.242 , 3.285	0.8547	2.202	0.953 , 5.087	0.0632
TNFRSF11B	2.097	0.456 , 9.653	0.3166	1.473	<0.001 , +Inf	0.8751
TNFSF10	1.242	0.697 , 2.213	0.446	1.011	0.573 , 1.783	0.9676
TNFSF11	2.147	0.730 , 6.315	0.1559	1.446	0.081 , 25.972	0.8005
TNFSF12	1.077	0.631 , 1.837	0.7777	0.769	0.468 , 1.262	0.2807
TNFSF13	1.443	0.918 , 2.267	0.1067	1.287	0.695 , 2.381	0.3752
TNFSF13B	1.327	0.833 , 2.116	0.2222	1.055	0.656 , 1.696	0.8173
TNFSF14	2.234	1.012 , 4.931	0.047	1.031	0.480 , 2.217	0.9333
TNFSF4	2.61	1.261 , 5.401	0.0129	1.663	0.703 , 3.937	0.2057
TNFSF8	1.824	1.081 , 3.079	0.0264	1.18	0.792 , 1.757	0.3961

TXLNA	1.246	0.826 , 1.878	0.276	0.883	0.661 , 1.180	0.3807
VEGFA	1.906	0.764 , 4.755	0.1555	1.902	0.170 , 21.291	0.598
