# Supplementary material for: Gene Expression Profile of Human Cytokines in Response to Burkholderia pseudomallei Infection
Source: mSphere. 2017 Apr 19;2(2):e00121-17. doi: 10.1128/mSphere.00121-17 (PMC5397567; doi:10.1128/mSphere.00121-17)
Supplement: TABLE S7 [file sph002172268st7.pdf]

Table S7

	melioidosis fever ≤ 15 days vs sepsis			melioidosis fever >15 days vs sepsis		
**Gene Target**	**Relative Expression Ratio**	**Confidence Limit**	**P-Value**	**Relative Expression Ratio**	**Confidence Limit**	**P-Value**
ADIPOQ	0.369	0.019 , 7.052	0.4256	3.038	0.384 , 24.000	0.271
BMP1	0.903	0.598 , 1.363	0.6009	1.341	0.903 , 1.991	0.1378
BMP2	0.543	0.282 , 1.045	0.0641	3.011	1.099 , 8.247	0.0333
BMP3	2.267	0.463 , 11.112	0.2541	6.494	2.715 , 15.538	0.0002
BMP4	4.117	0.428 , 39.595	0.1721	25.416	1.682 , 383.961	0.0231
BMP5	4.304	0.376 , 49.275	0.1862	3.259	0.478 , 22.208	0.2141
BMP6	2.639	0.929 , 7.500	0.0657	2.809	1.204 , 6.552	0.0201
BMP7	0.666	0.068 , 6.529	0.6871	1.504	0.099 , 22.853	0.7606
CD40LG	2.286	0.905 , 5.774	0.0759	2.272	0.972 , 5.310	0.057
CD70	1.407	0.647 , 3.064	0.3596	1.743	0.893 , 3.402	0.0946
CNTF	0.898	0.452 , 1.783	0.7168	2.758	1.098 , 6.927	0.0323
CSF1	1.013	0.383 , 2.681	0.9749	3.064	1.820 , 5.158	0.0002
CSF2	0.817	0.126 , 5.287	0.8138	2.992	0.741 , 12.087	0.1167
CSF3	0.591	0.097 , 3.613	0.4793	2.971	1.253 , 7.041	0.0154
FAM3B	1.51	0.312 , 7.300	0.5757	3.812	0.745 , 19.495	0.1026
FASLG	1.202	0.318 , 4.551	0.7684	2.173	0.717 , 6.581	0.1539
FIGF	1.765	0.727 , 4.290	0.1867	4.728	1.681 , 13.300	0.0046
GDF2				11.112	1.105 , 111.704	0.0421

GDF5	0.45	0.149 , 1.359	0.1384	4.427	1.068 , 18.350	0.0412
GDF9	1.451	0.527 , 3.995	0.426	1.212	0.462 , 3.181	0.6858
IFNA1	1.834	0.537 , 6.266	0.2902	4.722	1.440 , 15.482	0.0123
IFNA2				17.777	0.046 , +Inf	0.2395
IFNA4	3.704	0.070 , 196.370	0.3912	6.934	0.858 , 56.016	0.0667
IFNA5	0.082	0.004 , 1.892	0.0926	0.313	0.075 , 1.313	0.1021
IFNB1	1.322	0.452 , 3.866	0.5819	4.001	1.196 , 13.384	0.0262
IFNG	0.833	0.214 , 3.247	0.7697	3.202	1.149 , 8.925	0.0282
IL10	0.209	0.030 , 1.445	0.0934	1.173	0.518 , 2.655	0.6897
IL11	0.854	0.162 , 4.491	0.8215	1.322	0.447 , 3.912	0.5987
IL12A	0.953	0.353 , 2.576	0.9188	1.749	0.719 , 4.254	0.1976
IL12B	1.198	0.311 , 4.612	0.7685	2.974	0.952 , 9.295	0.0601
IL13	1.486	0.346 , 6.387	0.5457	2.909	0.938 , 9.017	0.063
IL15	0.72	0.548 , 0.946	0.0219	1.455	1.044 , 2.027	0.0281
IL16	1.495	0.788 , 2.836	0.195	2.006	1.208 , 3.333	0.0105
IL17A	3.148	0.471 , 21.029	0.1867	27.078	3.920 , 187.046	0.0022
IL17B	2.004	0.874 , 4.596	0.0903	3.219	1.499 , 6.915	0.004
IL17C	1.691	0.801 , 3.572	0.147	1.607	0.701 , 3.686	0.2519
IL18	0.914	0.491 , 1.700	0.7536	0.904	0.563 , 1.453	0.6596
IL19	0.793	0.240 , 2.618	0.6272	2.103	0.720 , 6.145	0.1626
IL1A	0.286	0.110 , 0.745	0.0163	0.419	0.104 , 1.691	0.21

IL1B	0.087	0.029 , 0.261	0.0004	0.981	0.330 , 2.920	0.9717
IL1RN	0.544	0.259 , 1.141	0.0905	2.306	1.455 , 3.656	0.0009
IL2	1.622	0.515 , 5.113	0.3762	1.321	0.407 , 4.287	0.6237
IL20	1.622	0.169 , 15.597	0.6414	3.31	0.676 , 16.207	0.1297
IL21	0.357	0.128 , 0.995	0.049	0.871	0.300 , 2.527	0.7918
IL22	1.081	0.252 , 4.631	0.9046	3.86	1.504 , 9.905	0.0071
IL23A	1.31	0.656 , 2.618	0.4122	3.344	1.534 , 7.288	0.004
IL24	2.045	0.811 , 5.154	0.1182	3.274	1.273 , 8.421	0.0161
IL25	0.813	0.103 , 6.420	0.8163	2.865	0.700 , 11.718	0.1354
IL27	0.846	0.261 , 2.743	0.7497	4.204	1.490 , 11.861	0.0083
IL3	3.914	<0.001 , +Inf	0.4223	10.54	1.691 , 65.698	0.0143
IL4	0.673	0.043 , 10.599	0.7223	11.479	1.666 , 79.101	0.0159
IL5	0.975	0.271 , 3.509	0.9647	1.142	0.428 , 3.044	0.7828
IL6	0.358	0.133 , 0.966	0.0437	2.114	0.739 , 6.050	0.1508
IL7	0.879	0.418 , 1.848	0.6952	1.688	0.871 , 3.270	0.1154
IL8	0.1	0.026 , 0.381	0.0047	0.858	0.333 , 2.213	0.7431
IL9	3.524	0.401 , 30.949	0.2216	2.626	0.298 , 23.137	0.3425
INHA	0.303	0.027 , 3.464	0.2965	13.059	1.647 , 103.563	0.0188
INHBA	1.363	0.340 , 5.466	0.6376	6.396	1.608 , 25.439	0.0115
LEFTY2	1.385	0.486 , 3.951	0.511	1.67	0.639 , 4.366	0.2791
LIF	0.112	0.028 , 0.458	0.0103	2.34	0.814 , 6.726	0.1097

LTA	1.061	0.572 , 1.971	0.8372	1.669	0.765 , 3.639	0.1884
LTB	1.354	0.667 , 2.747	0.3698	1.981	0.950 , 4.131	0.0662
MSTN	1.293	0.114 , 14.656	0.8111	2.454	0.539 , 11.173	0.2313
NODAL	0.937	0.439 , 2.000	0.8529	1.389	0.741 , 2.603	0.2876
OSM	0.308	0.128 , 0.743	0.0141	2.493	1.201 , 5.173	0.0162
PDGFA	2.017	0.663 , 6.135	0.1886	2.395	1.072 , 5.352	0.0349
SPP1	1.325	0.219 , 8.022	0.7353	1.994	0.390 , 10.212	0.3923
TGFA	0.446	0.170 , 1.172	0.0866	1.484	0.719 , 3.063	0.2719
TGFB1	1.37	0.902 , 2.081	0.1189	2.197	1.416 , 3.408	0.001
TGFB2	5.381	1.008 , 28.733	0.0491	3.339	0.678 , 16.456	0.1266
TGFB3	1.264	0.563 , 2.840	0.5425	1.985	0.879 , 4.482	0.0943
THPO	1.522	0.540 , 4.290	0.3886	5.241	1.102 , 24.928	0.0384
TNF	0.225	0.053 , 0.943	0.0425	1.269	0.358 , 4.500	0.6942
TNFRSF11B	1.231	0.080 , 18.920	0.8546	0.884	0.142 , 5.504	0.8891
TNFSF10	0.886	0.313 , 2.507	0.7809	1.568	0.958 , 2.566	0.0721
TNFSF11	1.133	0.474 , 2.710	0.7593	1.601	0.541 , 4.739	0.3795
TNFSF12	1.342	0.962 , 1.872	0.0789	1.182	0.732 , 1.910	0.4815
TNFSF13	0.964	0.671 , 1.385	0.8292	1.372	0.879 , 2.142	0.1562
TNFSF13B	0.657	0.310 , 1.393	0.2163	1.647	1.092 , 2.485	0.0191
TNFSF14	1.831	0.827 , 4.056	0.1224	2.497	1.173 , 5.315	0.0196
TNFSF4	1.565	0.665 , 3.680	0.2757	2.588	1.265 , 5.293	0.0125

TNFSF8	1.5	0.872 , 2.580	0.1295	1.634	0.999 , 2.672	0.0503
TXLNA	1.001	0.564 , 1.779	0.9958	1.274	0.843 , 1.923	0.2345
VEGFA	0.573	0.145 , 2.269	0.3477	1.443	0.634 , 3.288	0.3621
