# Supplementary material for: Gene Expression Profile of Human Cytokines in Response to Burkholderia pseudomallei Infection
Source: mSphere. 2017 Apr 19;2(2):e00121-17. doi: 10.1128/mSphere.00121-17 (PMC5397567; doi:10.1128/mSphere.00121-17)
Supplement: TABLE S8 [file sph002172268st8.pdf]

Table S8

	melioidosis ≤ 15 days antibiotics vs sepsis			melioidosis >15 days antibiotics vs sepsis		
Gene Target	Relative Expression Ratio	Confidence Limit	P-Value	Relative Expression Ratio	Confidence Limit	P-Value
ADIPOQ	2.651	0.328 , 21.428	0.3348	1.223	0.029 , 51.984	0.9008
BMP1	1.143	0.753 , 1.735	0.5116	1.489	0.881 , 2.516	0.1259
BMP2	1.805	0.504 , 6.459	0.3348	3.491	0.662 , 18.400	0.1216
BMP3	3.295	1.352 , 8.027	0.011	9.193	1.895 , 44.606	0.0109
BMP4	7.247	0.588 , 89.374	0.1095	48.057	0.232 , +Inf	0.1176
BMP5	3.709	0.421 , 32.686	0.2201	3.484	0.269 , 45.193	0.2995
BMP6	2.708	1.133 , 6.469	0.0275	3.683	1.285 , 10.551	0.0184
BMP7	0.765	0.089 , 6.534	0.7957	0.299	0.016 , 5.444	0.3831
CD40LG	2.09	0.878 , 4.974	0.0901	1.917	0.739 , 4.973	0.1669
CD70	1.614	0.828 , 3.149	0.1427	1.354	0.645 , 2.838	0.3963
CNTF	1.512	0.787 , 2.904	0.2004	5.17	0.480 , 55.737	0.1473
CSF1	2.027	1.120 , 3.669	0.0217	3.423	1.163 , 10.075	0.0301
CSF2	1.338	0.362 , 4.945	0.6397	4.569	0.425 , 49.169	0.1877
CSF3	1.484	0.608 , 3.624	0.3644	2.593	0.306 , 21.979	0.3305
FAM3B	2.314	0.540 , 9.920	0.2353	8.173	0.835 , 80.009	0.0676
FASLG	1.485	0.477 , 4.628	0.468	2.087	0.589 , 7.394	0.2353
FIGF	2.656	1.062 , 6.646	0.0379	4.345	0.471 , 40.037	0.1668
GDF2	9.652	1.240 , 75.129	0.0338	3.663	<0.001 , +Inf	0.7103

GDF5	2.826	0.391 , 20.437	0.2763	2.09	0.569 , 7.682	0.2447
GDF9	0.897	0.270 , 2.974	0.8507	2.363	0.805 , 6.934	0.1066
IFNA1	3.681	1.112 , 12.181	0.0343	3.803	0.281 , 51.495	0.268
IFNA2	24.904	0.037 , +Inf	0.2574	59.852	0.076 , +Inf	0.2168
IFNA4	4.095	0.569 , 29.492	0.1469	1.656	<0.001 , +Inf	0.7626
IFNA5	0.123	0.023 , 0.648	0.0183	0.529	0.060 , 4.630	0.4745
IFNB1	2.001	0.706 , 5.673	0.1772	2.622	0.621 , 11.064	0.1701
IFNG	1.588	0.553 , 4.560	0.37	3.502	0.789 , 15.532	0.0923
IL10	0.578	0.209 , 1.602	0.277	1.662	0.435 , 6.355	0.4227
IL11	1.734	0.607 , 4.950	0.2844	1.075	0.174 , 6.664	0.93
IL12A	1.526	0.608 , 3.832	0.3434	1.554	0.516 , 4.680	0.4071
IL12B	1.993	0.684 , 5.806	0.1939	2.743	0.314 , 23.969	0.3218
IL13	1.739	0.648 , 4.662	0.2479	2.261	0.248 , 20.597	0.4185
IL15	0.999	0.716 , 1.393	0.9932	1.642	0.894 , 3.017	0.0989
IL16	1.709	0.994 , 2.938	0.0522	1.878	1.074 , 3.284	0.0296
IL17A	33.758	3.476 , 327.839	0.0052	5.645	1.322 , 24.105	0.0253
IL17B	2.353	0.964 , 5.744	0.0592	3.344	0.982 , 11.385	0.0528
IL17C	1.659	0.696 , 3.957	0.2383	1.559	0.358 , 6.781	0.5096
IL18	0.836	0.509 , 1.374	0.4585	1.137	0.616 , 2.099	0.6621
IL19	1.058	0.333 , 3.361	0.9172	2.227	0.363 , 13.647	0.3293
IL1A	0.265	0.050 , 1.388	0.1075	0.492	0.059 , 4.105	0.4665

IL1B	0.474	0.134 , 1.674	0.2329	1.205	0.205 , 7.087	0.8215
IL1RN	1.164	0.674 , 2.011	0.5679	3.449	1.354 , 8.788	0.0154
IL2	1.644	0.529 , 5.107	0.3639	0.507	0.099 , 2.594	0.3702
IL20	2.192	0.450 , 10.676	0.3077	7.814	0.614 , 99.378	0.0994
IL21	0.48	0.179 , 1.290	0.1377	0.717	0.074 , 6.976	0.7408
IL22	2.048	0.802 , 5.232	0.1262	3.57	0.814 , 15.655	0.0851
IL23A	2.286	1.100 , 4.751	0.029	4.102	1.011 , 16.646	0.0486
IL24	2.758	1.142 , 6.659	0.0265	3.964	1.098 , 14.318	0.0375
IL25	2.214	0.535 , 9.168	0.2558	3.075	0.248 , 38.119	0.3317
IL27	1.763	0.730 , 4.255	0.1957	8.383	0.786 , 89.362	0.0721
IL3	5.41	0.945 , 30.966	0.0568	3.52	0.447 , 27.756	0.1894
IL4	1.563	0.459 , 5.319	0.4515	63.393	0.527 , +Inf	0.0788
IL5	1.006	0.415 , 2.438	0.9887	0.914	0.138 , 6.060	0.9156
IL6	1.046	0.333 , 3.290	0.9353	2.89	0.714 , 11.699	0.1263
IL7	1.058	0.552 , 2.027	0.857	2.689	0.789 , 9.161	0.1007
IL8	0.386	0.125 , 1.194	0.0942	1.83	0.478 , 7.009	0.3441
IL9	3.103	0.359 , 26.849	0.2645	1.967	0.217 , 17.856	0.4994
INHA	3.33	0.281 , 39.524	0.3179	13.205	0.630 , 276.872	0.0859
INHBA	4.307	1.033 , 17.951	0.0455	5.869	0.886 , 38.868	0.0642
LEFTY2	1.968	0.768 , 5.045	0.148	1.404	0.303 , 6.497	0.6337
LIF	1.274	0.265 , 6.124	0.7457	2.179	0.274 , 17.345	0.4126

LTA	1.665	0.809 , 3.426	0.1559	1.08	0.237 , 4.926	0.9121
LTB	1.829	0.868 , 3.854	0.1056	1.827	0.664 , 5.024	0.2226
MSTN	2.348	0.550 , 10.016	0.2314	3.065	0.192 , 48.811	0.3847
NODAL	1.229	0.674 , 2.244	0.478	1.037	0.347 , 3.103	0.9406
OSM	1.232	0.472 , 3.218	0.6552	2.514	0.750 , 8.424	0.1209
PDGFA	2.363	1.010 , 5.530	0.0476	3.278	1.224 , 8.781	0.0213
SPP1	2.185	0.512 , 9.330	0.2738	3.989	0.101 , 158.118	0.3983
TGFA	0.778	0.343 , 1.763	0.5238	2.159	0.616 , 7.574	0.1959
TGFB1	1.873	1.165 , 3.010	0.0124	2.588	1.042 , 6.425	0.0425
TGFB2	3.463	0.715 , 16.778	0.1123	7.729	1.516 , 39.406	0.0177
TGFB3	1.517	0.692 , 3.329	0.2783	2.75	0.707 , 10.690	0.1298
THPO	5.708	1.414 , 23.039	0.0173	3.269	0.093 , 114.838	0.4308
TNF	0.728	0.180 , 2.939	0.6393	1.446	0.291 , 7.182	0.6315
TNFRSF11B	1.134	0.149 , 8.626	0.8965	1.235	0.138 , 11.080	0.8299
TNFSF10	1.186	0.694 , 2.026	0.5156	1.616	0.697 , 3.747	0.2322
TNFSF11	1.194	0.418 , 3.416	0.7278	2.445	0.640 , 9.343	0.1707
TNFSF12	1.512	1.072 , 2.134	0.0211	0.791	0.259 , 2.413	0.6406
TNFSF13	1.346	0.841 , 2.154	0.203	1.462	0.579 , 3.694	0.3576
TNFSF13B	1.123	0.681 , 1.853	0.6337	2.041	0.942 , 4.422	0.0666
TNFSF14	2.18	1.061 , 4.477	0.0352	1.991	0.486 , 8.149	0.3012
TNFSF4	2.386	1.144 , 4.977	0.0232	2.768	1.055 , 7.265	0.04

TNFSF8	1.469	0.889 , 2.429	0.1251	1.882	0.923 , 3.840	0.0771
TXLNA	1.255	0.813 , 1.939	0.2881	1.305	0.724 , 2.352	0.3468
VEGFA	1.191	0.420 , 3.380	0.7269	1.647	0.659 , 4.117	0.2574
