# Supplementary material for: Gene Expression Profile of Human Cytokines in Response to Burkholderia pseudomallei Infection
Source: mSphere. 2017 Apr 19;2(2):e00121-17. doi: 10.1128/mSphere.00121-17 (PMC5397567; doi:10.1128/mSphere.00121-17)
Supplement: TABLE S9 [file sph002172268st9.pdf]

Table S9

**Gene Target**	**Relative Expression Ratio**	**Confidence Limit**	**P-Value**
ADIPOQ	5.346	0.120 , 238.898	0.2979
BMP1	1.002	0.627 , 1.601	0.9944
BMP2	1.302	0.303 , 5.591	0.7049
BMP3	1.395	0.427 , 4.564	0.5642
BMP4	0.426	0.008 , 21.402	0.6187
BMP5	0.092	<0.001 , 100.995	0.2350
BMP6	0.980	0.506 , 1.897	0.9496
BMP7	3.452	0.028 , 423.626	0.5819
CD40LG	1.141	0.596 , 2.184	0.6745
CD70	1.126	0.724 , 1.753	0.5675
CNTF	2.111	0.557 , 8.001	0.2582
CSF1	1.695	0.842 , 3.413	0.1311
CSF2	2.802	0.696 , 11.279	0.1384
CSF3	1.995	0.401 , 9.924	0.3749
FAM3B	12.138	2.487 , 59.237	0.0048
FASLG	1.229	0.522 , 2.890	0.6169
FIGF	1.414	0.318 , 6.279	0.6319
GDF2	0.951	<0.001 , +Inf	0.9824
GDF5	0.249	0.002 , 35.238	0.4566
GDF9	0.837	0.265 , 2.645	0.7506
IFNA1	0.788	0.136 , 4.571	0.7760
IFNA2	0.004	<0.001 , 0.967	0.0487
IFNA4	0.453	0.029 , 6.952	0.5193
IFNA5	0.999	0.066 , 15.048	0.9991
IFNB1	2.459	0.743 , 8.140	0.1322
IFNG	2.269	0.780 , 6.601	0.1253
IL10	3.561	1.098 , 11.543	0.0361
IL11	1.518	0.243 , 9.476	0.6129
IL12A	1.030	0.469 , 2.263	0.9356
IL12B	1.526	0.333 , 6.988	0.5668
IL13	2.790	0.741 , 10.501	0.1223
IL15	1.233	0.732 , 2.078	0.4060
IL16	0.943	0.600 , 1.481	0.7864
IL17A	0.660	0.003 , 144.868	0.8416
IL17B	1.476	0.360 , 6.050	0.5543
IL17C	1.596	0.428 , 5.951	0.4609
IL18	0.955	0.584 , 1.561	0.8445
IL19	1.767	0.368 , 8.486	0.4443
IL1A	0.654	0.117 , 3.667	0.6107
IL1B	2.273	0.481 , 10.738	0.2782
IL1RN	1.240	0.556 , 2.765	0.5822

IL2	0.945	0.382 , 2.335	0.8962
IL20	0.830	0.220 , 3.131	0.7720
IL21	1.266	0.330 , 4.862	0.7195
IL22	2.454	0.764 , 7.880	0.1226
IL23A	1.921	0.882 , 4.185	0.0961
IL24	1.838	0.480 , 7.042	0.3419
IL25	2.671	0.189 , 37.714	0.3946
IL27	1.586	0.371 , 6.782	0.5173
IL3	0.075	0.002 , 3.188	0.1456
IL4	6.345	0.500 , 80.440	0.1448
IL5	2.678	0.796 , 9.004	0.1038
IL6	3.307	1.278 , 8.560	0.0163
IL7	1.839	0.801 , 4.226	0.1429
IL8	4.170	0.992 , 17.522	0.0511
IL9	0.506	0.173 , 1.475	0.1813
INHA	1.944	<0.001 , +Inf	0.7413
INHBA	1.472	0.292 , 7.417	0.6099
LEFTY2	1.575	0.534 , 4.643	0.3853
LIF	1.475	0.219 , 9.951	0.6615
LTA	1.240	0.547 , 2.810	0.5917
LTB	1.095	0.511 , 2.348	0.8023
MSTN	1.195	0.246 , 5.812	0.8168
NODAL	1.616	0.851 , 3.069	0.1334
OSM	2.346	0.705 , 7.801	0.1514
PDGFA	1.221	0.568 , 2.626	0.5925
SPP1	0.927	0.155 , 5.559	0.9307
TGFA	2.591	0.978 , 6.860	0.0548
TGFB1	1.146	0.514 , 2.557	0.7197
TGFB2	1.847	0.647 , 5.273	0.2374
TGFB3	1.280	0.533 , 3.071	0.5608
THPO	4.537	0.058 , 357.591	0.3820
TNF	0.943	0.236 , 3.764	0.9286
TNFRSF11B	9.277	0.480 , 179.439	0.1172
TNFSF10	0.702	0.382 , 1.290	0.2410
TNFSF11	2.649	0.715 , 9.813	0.1350
TNFSF12	0.712	0.399 , 1.270	0.2374
TNFSF13	1.391	0.741 , 2.610	0.2835
TNFSF13B	0.893	0.432 , 1.848	0.7454
TNFSF14	0.861	0.344 , 2.154	0.7357
TNFSF4	1.356	0.682 , 2.696	0.3618
TNFSF8	1.424	0.977 , 2.075	0.0648
TXLNA	1.072	0.638 , 1.801	0.7776
VEGFA	2.973	1.177 , 7.513	0.0235
